# Purinergic GPCR-integrin interactions drive pancreatic cancer cell invasion

**DOI:** 10.7554/eLife.86971

**Published:** 2023-03-21

**Authors:** Elena Tomas Bort, Megan D Joseph, Qiaoying Wang, Edward P Carter, Nicolas J Roth, Jessica Gibson, Ariana Samadi, Hemant M Kocher, Sabrina Simoncelli, Peter J McCormick, Richard P Grose

**Affiliations:** 1 Centre for Tumour Biology, Barts Cancer Institute, Queen Mary University of London London United Kingdom; 2 Centre for Endocrinology, William Harvey Research Institute, Queen Mary University of London London United Kingdom; 3 London Centre for Nanotechnology, University College London London United Kingdom; 4 Department of Chemistry, University College London London United Kingdom; Memorial Sloan Kettering Cancer Center United States; Memorial Sloan Kettering Cancer Center United States

**Keywords:** pancreatic cancer, purinergic signalling, invasion, DNA-PAINT, 3D modelling, Human

## Abstract

Pancreatic ductal adenocarcinoma (PDAC) continues to show no improvement in survival rates. One aspect of PDAC is elevated ATP levels, pointing to the purinergic axis as a potential attractive therapeutic target. Mediated in part by highly druggable extracellular proteins, this axis plays essential roles in fibrosis, inflammation response, and immune function. Analyzing the main members of the PDAC extracellular purinome using publicly available databases discerned which members may impact patient survival. *P2RY2* presents as the purinergic gene with the strongest association with hypoxia, the highest cancer cell-specific expression, and the strongest impact on overall survival. Invasion assays using a 3D spheroid model revealed P2Y_2_ to be critical in facilitating invasion driven by extracellular ATP. Using genetic modification and pharmacological strategies, we demonstrate mechanistically that this ATP-driven invasion requires direct protein-protein interactions between P2Y_2_ and αV integrins. DNA-PAINT super-resolution fluorescence microscopy reveals that P2Y_2_ regulates the amount and distribution of integrin αV in the plasma membrane. Moreover, receptor-integrin interactions were required for effective downstream signaling, leading to cancer cell invasion. This work elucidates a novel GPCR-integrin interaction in cancer invasion, highlighting its potential for therapeutic targeting.

## Introduction

PDAC, which accounts for 90% of diagnosed pancreatic cancer cases, has the lowest survival rate of all common solid malignancies. Surgery is the only potentially curative treatment, yet more than 80% of patients present with unresectable tumors (Kocher, 2023). Consequently, most patients survive less than six months after diagnosis, resulting in a five year survival rate of less than 5% when accounting for all disease stages (Bengtsson et al., 2020; Kocher, 2023). Despite continued efforts, this statistic has improved minimally in the past 50 years. Due to increasing incidence, late detection, and lack of effective therapies, pancreatic cancer is predicted to be the second most common cause of cancer-related deaths by 2040 (Rahib et al., 2021).

Failure to significantly improve clinical management is mainly a result of chemoresistance (Neuzillet et al., 2017), thus it is of vital importance to find new therapeutics that can improve patient survival. PDAC is characterized by its desmoplastic stroma, with dense fibrosis leading to impaired vascularisation and high levels of hypoxia (Koong et al., 2000; Di Maggio, 2016). Lack of oxygen leads to cellular stress and death, resulting in the release of purines such as ATP and adenosine into the tumor microenvironment (Forrester and Williams, 1977; Pellegatti et al., 2008). Extracellular ATP concentration in PDAC is 200-fold more than in normal tissue (Hu et al., 2019), suggesting that purinergic signaling could represent an effective therapeutic target in pancreatic cancer.

The proteins underpinning purinergic signaling comprise several highly druggable membrane proteins involved in the regulation of extracellular purines, mainly ATP and adenosine (Burnstock and Novak, 2012; Boison and Yegutkin, 2019; Yu et al., 2021). Extracellular ATP is known to promote inflammation (Kurashima et al., 2012), growth (Ko et al., 2012), and cell movement (Martínez-Ramírez et al., 2016). Contrastingly, adenosine is anti-inflammatory and promotes immunosuppression (Schneider et al., 2021). There are ongoing clinical trials in several cancers, including PDAC, for drugs targeting the ectonucleotidase CD73 (NCT03454451, NCT03454451) and adenosine receptor 2 A (NCT03454451) in combination with PD-1 checkpoint inhibitors and/or chemotherapy. However, a Phase II multi-cancer study evaluating an anti-CD73 and anti-PD-L1 combination was withdrawn due to minimal overall clinical activity (NCT04262388). This suggests that the oncogenic impact of purinergic signaling may act via pathways other than immunosuppression and highlights the need for the further mechanistic understanding of purinergic signaling in PDAC to exploit its full therapeutic potential.

Here, we combine bioinformatic, genetic, and drug-based approaches to identify a novel mechanism mediating ATP-driven invasion, uncovering a new therapeutic target in PDAC, a cancer of unmet clinical need. Beginning with an in-depth in silico analysis of the purinergic signaling transcriptome in PDAC, using publicly available patient and cell line databases, we build on bioinformatic data associating the purinergic receptor P2Y_2_ with PDAC. After validating the expression of P2Y_2_ in human PDAC, we focus on identifying the function of the receptor in cancer cells. In vitro data underline the importance of P2Y_2_ as a strong invasive driver, using a 3D physio-mimetic model of invasion. Finally, using a super-resolution imaging technique, DNA-PAINT, we characterize the behavior of P2Y_2_ in the membrane at the single molecule level, demonstrating the nanoscale distribution and interaction of this receptor with RGD-binding integrins in promoting pancreatic cancer invasion.

## Results

### The PDAC extracellular purinome associates with patient survival, hypoxia score and cell phenotype

**Figure 1.**
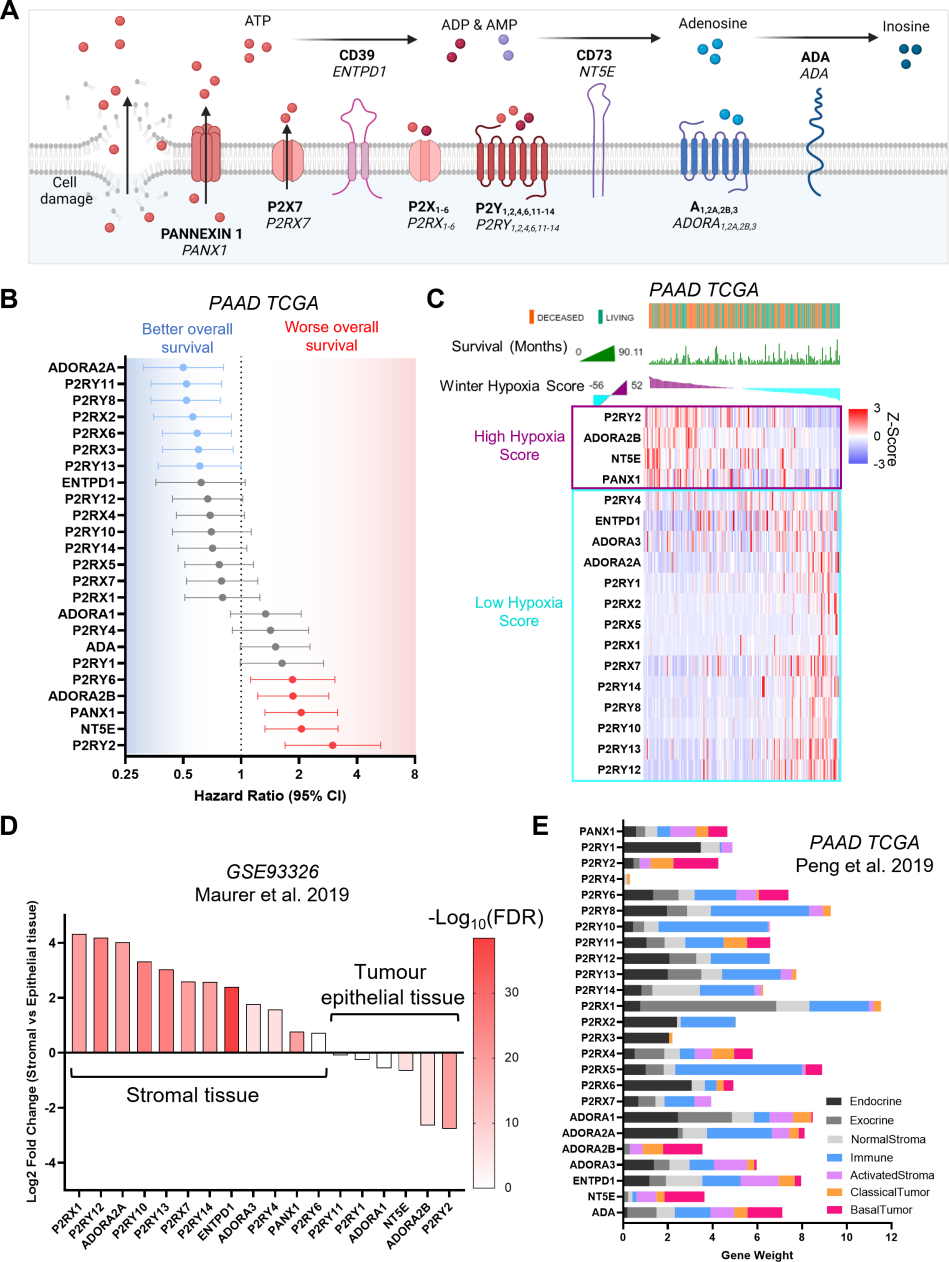
Characterization of purinergic signaling in pancreatic adenocarcinoma. (**A**) Purinergic signaling proteins and gene names. (**B**) Hazard ratios of overall survival were calculated using KMPlot and the pancreatic adenocarcinoma (PAAD) The Cancer Genome Atlas (TCGA) cohort (n=177) for different purinergic genes. Statistically significant hazard ratios (log-rank p-value) are highlighted in red for worse survival and in blue for better survival. (**C**) Heatmap of purinergic genes significantly correlated (*q*<0.05) to high (purple) or low (light blue) Winter hypoxia scores in the PAAD TCGA data set. Overall survival status and overall survival in months are shown at the top, and samples are ranked using the Winter Hypoxia score (Generated with cBioPortal). (**D**) Differential expression analysis of 60 paired stromal and tumor tissue microdissections (GSE93326) showing significantly differentially expressed purinergic genes in stromal or tumor epithelial tissue. (**E**) Gene weights for purinergic genes representing the relevance of each gene to each cell type compartment, obtained from DECODER PDAC TCGA deconvolution analysis.

The extracellular purinome encompasses 23 main surface proteins, including pannexin 1, P2X ion channels, ectonucleotidases, and the P2Y, and adenosine GPCRs (Di Virgilio et al., 2018; Figure 1A). Interrogating public databases, we determined which purinergic signaling genes significantly impact pancreatic cancer survival. First, we examined the pancreatic adenocarcinoma (PAAD) database from The Cancer Genome Atlas (TCGA; n=177 patients), analyzing overall survival hazard ratios based on purinergic signaling gene expression (Figure 1B). Expression of five purinergic genes correlated with decreased patient survival, with high *P2RY2* expression being associated with the highest hazard ratio (2.99, 95%, CI: 1.69–5.31, log-rank p=8.5 × 10^–5^). We then examined the mutational profile and mRNA expression level of purinergic genes in patients. Using cBioPortal (Gao, 2013), we generated OncoPrints of purinergic signaling genes from PAAD TCGA samples (Figure 1—figure supplement 1A), observing few genetic alterations in 0–3% of tumors and a heterogeneous percentage of tumors with high mRNA expression (z-score >1) for each purinergic gene. PDAC molecular subtypes associated with purinergic signaling genes were varied (Supplementary file 1). In the Bailey model, most genes were related to the immunogenic subtype except for *NT5E*, *ADORA2B*, *PANX1,* and *P2RY2*, which are related to squamous (Bailey et al., 2016). Collisson molecular subtyping showed several purinergic genes associated mostly with quasimesenchymal and exocrine subtypes (Collisson et al., 2011). The Moffitt subtypes were not strongly associated with purinergic genes except for *ADA*, *NT5E*, *P2RY6*, *P2RY2,* and *PANX1* associated with the Basal subtype (Moffitt et al., 2015).

**Figure 1—figure supplement 1.**
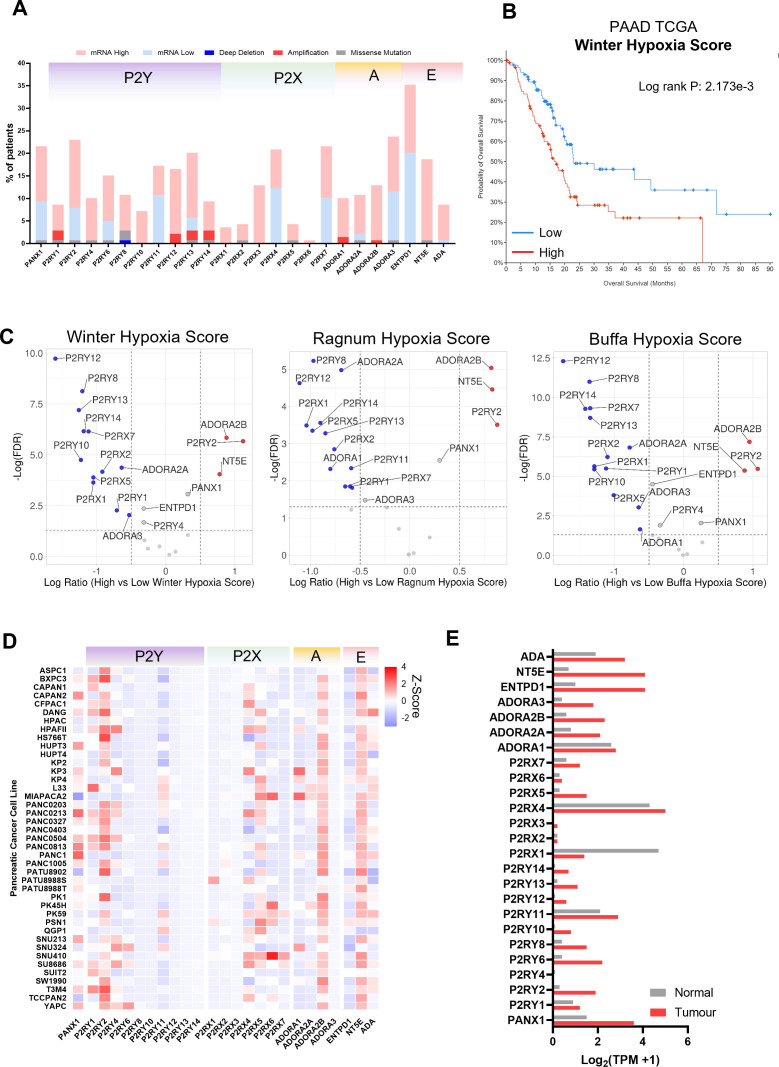
Characterization of purinergic genes in pancreatic adenocarcinoma. (**A**) Oncoprint from the pancreatic adenocarcinoma (PAAD) The Cancer Genome Atlas (TCGA) cohort was generated using cBioPortal. mRNA high and mRNA low represent Z-score values of >1 or < −1.(**B**) KMplot generated in cBioPortal for patients with high (red) vs low (blue) Winter hypoxia scores (**C**) Volcano plots for differential expression results of PAAD TCGA patient of high or low hypoxia scores using three different hypoxia signatures (Winter, Ragnum, and Buffa). (**D**) Heat map of purinergic mRNA expression data for different Pancreatic ductal adenocarcinoma (PDAC) cell lines from CCLE. (**E**) Comparison of normal versus tumor normalized transcripts per million (TPM) expression of purinergic genes. Data was obtained using GEPIA and PAAD TCGA and GTEx.

PDAC is known for its hypoxic environment (Koong et al., 2000; Yuen and Díaz, 2014), which is associated with worse overall survival (p=0.002, Figure 1—figure supplement 1B); hypoxia can lead to cellular stress and death, resulting in the increase of extracellular purines (Forrester and Williams, 1977). The Winter (Winter et al., 2007), Ragnum (Ragnum et al., 2015), and Buffa (Buffa et al., 2010) hypoxia scores were used to examine the correlation between the expression of purinergic genes and hypoxia in the PAAD TCGA database (Figure 1—figure supplement 1C). Samples were divided into low (n=88) or high (n=89) hypoxia score, using the median hypoxia score to perform a differential expression analysis. CD73 (*NT5E*), adenosine A2B receptor (*ADORA2B*), and P2Y_2_ (*P2RY2*) mRNA expression associated strongly with the high hypoxia score group for all three hypoxia scores (log_2_ ratio >0.5, FDR < 0.0001). P2Y_2_ had the highest log_2_ ratio in all hypoxia signatures compared to other purinergic genes. With a more extensive gene signature, the Winter hypoxia score (99 genes) allowed for a more comprehensive relative hypoxia ranking of tumor samples, compared to Ragnum (32 genes) and Buffa (52 genes) signatures. Hence, we used cBioPortal (Gao, 2013) to generate a transcriptomic heatmap of purinergic genes, ranked using the Winter hypoxia score and overlaid with overall survival data (Figure 1C). Taken together, these results show a direct correlation between Winter hypoxia score and decreased overall survival for high hypoxia score-related purinergic genes.

We hypothesized that genes related to high hypoxia scores would be expressed preferentially in the tumor cell compartment, as PDAC cells inhibit angiogenesis, causing hypo-vascularisation in the juxta-tumoral stroma (Di Maggio, 2016). Mining published RNA-seq data from 60 paired PDAC samples of stroma and tumor microdissections (GSE93326) (Maurer et al., 2019) and performing differential expression analysis, we observed that most genes related to high Winter hypoxia scores (*P2RY2*, *ADORA2B,* and *NT5E*) were expressed in the tumor epithelial tissue (Figure 1D), except for *PANX1*, encoding for pannexin 1, which is involved in cellular ATP release (Bao et al., 2004).

To elucidate the cell type-specific purinergic expression landscape, we used published data from TCGA PAAD compartment deconvolution, using DECODER (Peng et al., 2019) to plot purinergic gene weights for each cell type compartment (Figure 1E). The findings recapitulated the cell specificity data obtained from tumor microdissection analysis (Maurer et al., 2019; Figure 1D). Expression of purinergic genes in cancer cells was confirmed by plotting Z-scores of mRNA expression of PDAC cell lines from the cancer cell line encyclopedia (Ghandi et al., 2019) (CCLE; Figure 1—figure supplement 1D). Moreover, the expression of purinergic genes in normal tissue from the Genotype-Tissue Expression (GTEx) database compared to cancer tissue (PAAD TCGA) also mimicked the results found with DECODER (Figure 1—figure supplement 1E). *P2RY2*, encoding P2Y_2_ - a GPCR activated by ATP and UTP, was shown to be the purinergic gene most highly associated with cancer cell-specific expression in all our independent analyses (Figure 1D and E; Figure 1—figure supplement 1D, E). *P2RY2* additionally showed the strongest correlation with all hypoxia scores (Figure 1C; Figure 1—figure supplement 1C). Most importantly, of all purinergic genes, *P2RY2* expression had the biggest adverse impact on patient survival (Figure 1B). These independent in silico analyses encouraged us to explore the influence of P2Y_2_ on pancreatic cancer cell behavior.

### *P2RY2* is expressed in cancer cells and causes cytoskeletal changes

**Figure 2.**
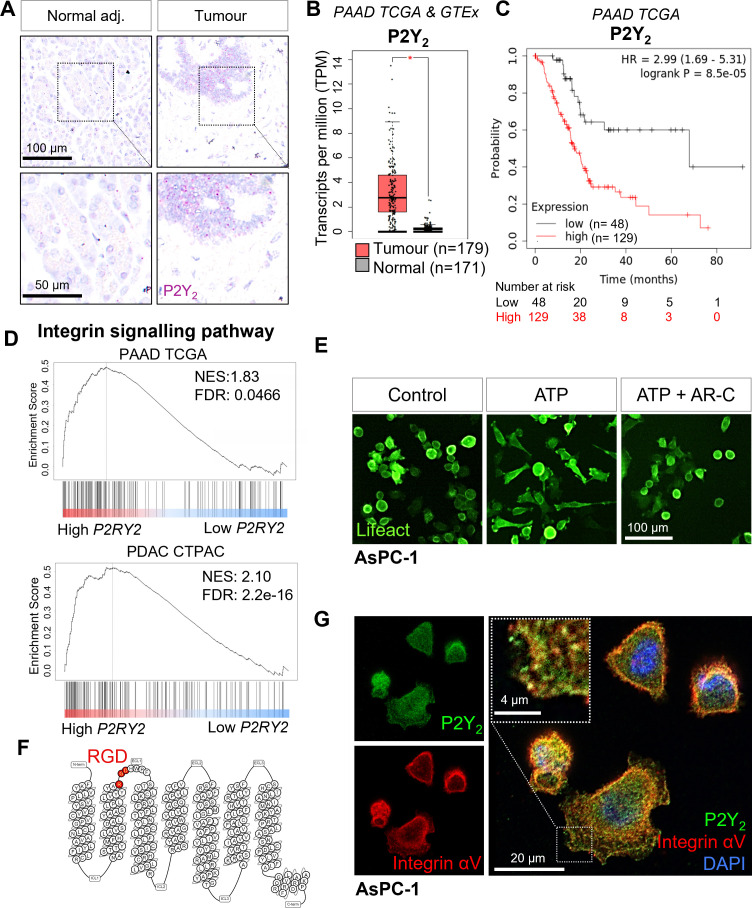
Expression of P2Y_2_ is specific to cancer cells, correlated with decreased overall survival in patients, and drives cytoskeletal rearrangements. (**A**) RNAscope in-situ hybridization of P2Y_2_ mRNA expression (magenta) in tumor and matching normal adjacent tissue. (**B**) P2Y_2_ mRNA expression in tumor (TCGA) and normal (GTEx) pancreatic tissue samples (*p<0.0001). Graph generated using GEPIA. (**C**) Kaplan-Meier plot comparing patients with high vs low expression of P2Y_2_ in the Pancreatic Adenocarcinoma (PAAD) The Cancer Genome Atlas (TCGA) cohort. Graph generated using KMplot. (**D**) Top result of a GSEA (performed with WebGestalt) of two different pancreatic adenocarcinoma patient cohorts (PAAD TCGA and PDAC CPTAC) for the PANTHER pathway functional database. (**E**) Incucyte images of the pancreatic cancer cell line AsPC-1 12 hr after treatment with 100 µM ATP alone or with 5 µM AR-C (P2Y_2_ antagonist). Cells are transduced with Lifeact to visualize f-actin (green). (**F**) Schematic of the amino acid sequence of P2Y_2_ showing an RGD motif in the first extracellular loop (image generated in http://gpcrdb.org/). (**G**) IF staining of P2Y_2_ (green), integrin αV (red), and DAPI (blue) in AsPC-1 cells showing colocalization of P2Y_2_ and integrin αV (yellow).

To validate our bioinformatic findings, based on microdissections from a 60 patient cohort (GSE93326) and from the deconvolution of 177 PAAD tissues from the TCGA, we performed RNAscope on human PDAC samples. This corroborated P2Y_2_ mRNA expression as being localized to the epithelial tumor cell compartment and not stroma, normal epithelium, or endocrine tissues (n=3, representative images of 2 different patients shown in Figure 2A and Figure 2—figure supplement 1A), matching our findings from larger publicly available cohorts, including P2Y_2_ IHC data from 264 patients in the Renji cohort (Hu et al., 2019). P2Y_2_ is known to be expressed at low levels in normal tissues but interestingly RNAscope did not detect this. This data suggests (1) the lower limits of the technique compounded by the challenge of RNA degradation in pancreatic tissue and (2) supports that in tumor tissue where it was detected there was indeed overexpression of P2Y_2_, in line with the bioinformatic data. Interrogating single-cell P2Y_2_ RNA expression in normal PDAC from https://www.proteinatlas.org/ (Karlsson et al., 2021), expression was found at low levels in several cells types, for example in endocrine cells and macrophages (Figure 2—figure supplement 1B). Using GEPIA (Tang et al., 2017), we analyzed PAAD TCGA and GTEx mRNA expression of tumor (n=179) and normal samples (n=171). Tumor samples expressed significantly higher (p<0.0001) P2Y_2_ mRNA levels compared to the normal pancreas (Figure 2B). Kaplan-Meier analysis from PAAD TCGA KMplot (Lánczky and Győrffy, 2021) showed a significant decrease in median overall survival in patients with high P2Y_2_ mRNA expression (median survival: 67.87 vs 17.27 months) (Figure 2C).

**Figure 2—figure supplement 1.**
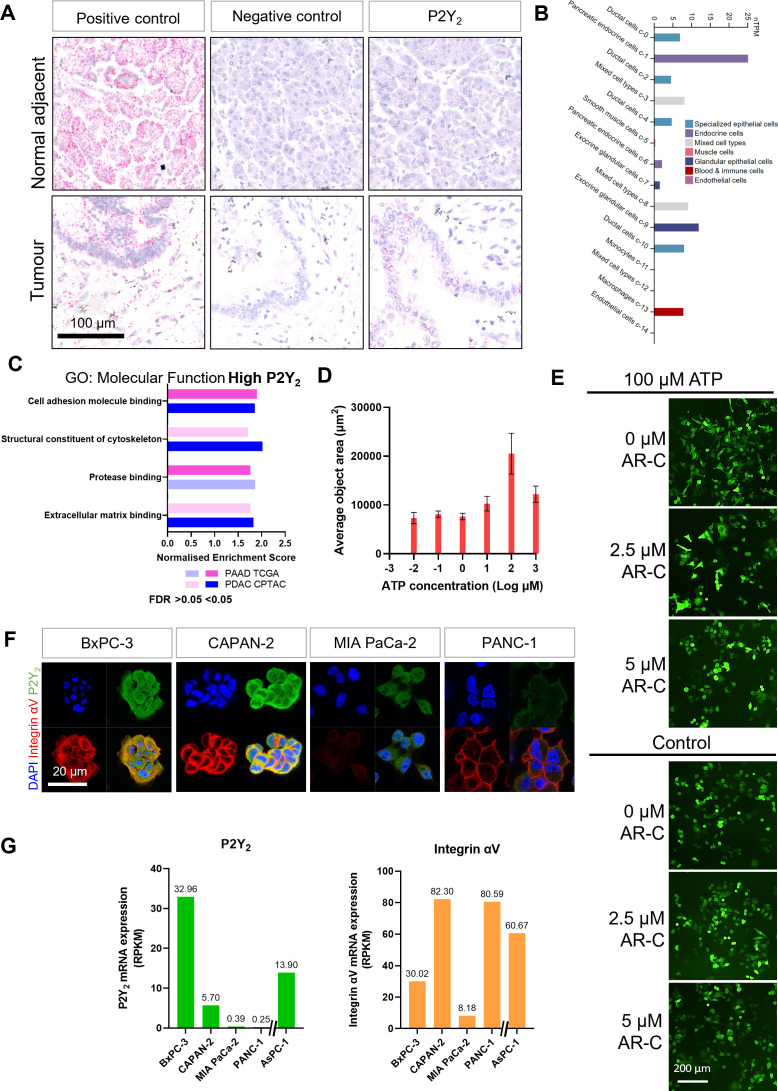
mRNA and protein expression of P2Y_2_ in pancreatic ductal adenocarcinoma (PDAC) cells. (**A**) RNAscope in-situ hybridization of a positive control (*PPIB*, Cyclophilin B), negative control (*DapB*), and P2Y_2_ mRNA expression in a PDAC tissue slide showing tumor and normal adjacent tissue. (**B**) Single-cell expression of P2Y_2_ in healthy pancreatic tissue from the Human Protein Atlas (https://www.proteinatlas.org/ENSG00000175591-P2RY2/single+cell+type/pancreas). (**C**) Top four results of a GSEA (performed with WebGestalt) of two different pancreatic adenocarcinoma patient cohorts (PAAD TCGA and PDAC CPTAC) for the ‘Molecular Function’ Gene Ontology (GO) functional database. (**D**) Incucyte analysis of average object area related to the average cell area of AsPC-1 cells at different concentrations of ATP (error bars show standard deviation). (**E**) Incucyte images of AsPC-1 cells with different concentrations of AR-C with or without ATP. (**F**) IF staining of four different PDAC cell lines showing various levels of P2Y_2_ (green) and integrin αV (red) protein expression. (**G**) The respective reads per kilobase of exon per million reads mapped (RPKM) from CCLE.

To predict P2Y_2_ function in PDAC, we performed gene set enrichment analysis (GSEA) of high vs low mRNA expressing P2Y_2_ tumor samples, divided by the median expression, for PAAD TCGA (n=177) and the PDAC Clinical Proteomic Tumor Analysis Consortium (CPTAC) (n=140) databases. The top gene set enriched in the PANTHER pathway database in both cohorts was the ‘integrin signaling pathway’ (Figure 2D). The top four enriched gene sets from the Gene Ontology ‘Molecular function’ functional database were associated with cell adhesion molecule binding, the cytoskeleton, protease binding, and extracellular matrix binding (Figure 2—figure supplement 1C). As preliminary validation of the GSEA results in vitro, we used the PDAC cell line AsPC-1, transduced with Lifeact, a peptide that fluorescently labels filamentous actin structures (Riedl et al., 2008), and monitored cell morphology using the Incucyte live-cell analysis system. Cells treated with ATP (100 µM) showed cytoskeletal rearrangements which were blocked by the selective P2Y_2_ antagonist AR-C118925XX (AR-C; 5 µM; Figure 2E; Muoboghare et al., 2019). Exposing cells to ATP at 100 µM resulted in the biggest change in cell area when testing six concentrations from 0.01 to 1000 µM (Figure 2—figure supplement 1D). ATP-driven morphological changes were fully reversed at 5 X (5 µM) the IC_50_ of AR-C (1 µM), while AR-C on its own had no effect on cell morphology (Figure 2—figure supplement 1E).

P2Y_2_ is the only P2Y GPCR possessing an RGD motif, located in the first extracellular loop (Figure 2F). P2Y_2_ has been shown to interact with αV integrins through this RGD motif (Erb et al., 2001), but the significance of this interaction has not been explored in cancer. Immunofluorescence (IF) showed colocalization of integrin αV and P2Y_2_ in the PDAC cell lines AsPC-1 as well as PDAC cell lines with strong epithelial morphology, BxPC-3 and CAPAN-2, while MIA PaCa-2 cells showed low expression of both proteins, and PANC-1 showed high integrin αV and low P2Y_2_, matching CCLE data (Figure 2G; Figure 2—figure supplement 1F, G). We hypothesized that P2Y_2,_ through its RGD motif, could engage αV integrins in cancer cells in the presence of ATP, leading to increased migration and invasion.

### Targeting P2Y_2_ and its RGD motif decreases ATP-driven invasion in PDAC cell lines

**Figure 3.**
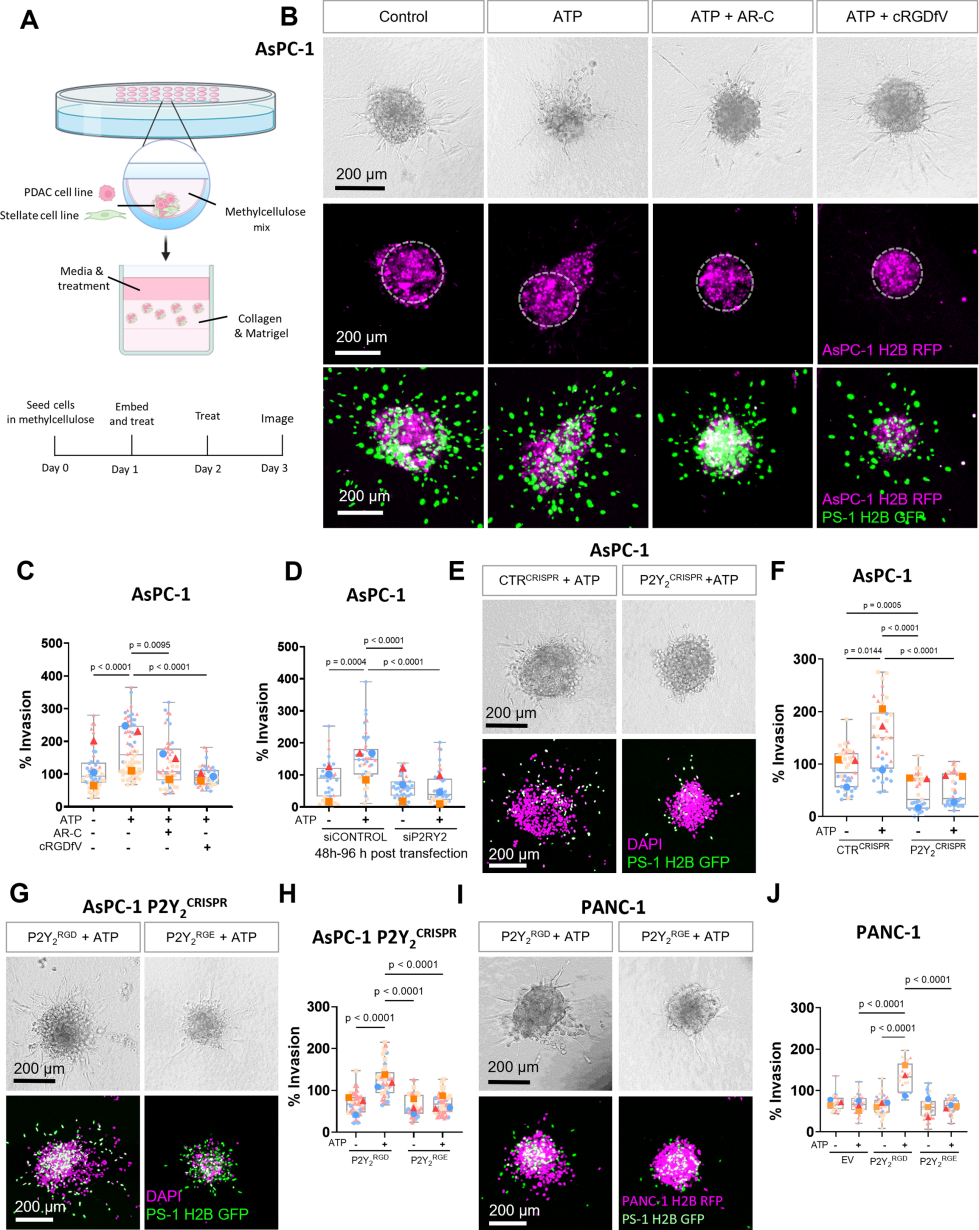
The RGD motif in P2Y_2_ is required for extracellular ATP-driven cancer cell invasion. (**A**) Schematic diagram of the hanging drop sphere model for 3D sphere invasion assays. (**B**) Bright field and fluorescent images of spheres formed using AsPC-1 cells (magenta) with a histone 2B (H2B) tagged with a red fluorescent protein (RFP) and the stellate cell line PS-1 (green) with H2B tagged with a green fluorescent protein (GFP). Middle panel shows AsPC-1 cells in spheres with a dotted line highlighting the central sphere area. Spheres were treated with vehicle control or 100 µM ATP alone or with 5 µM AR-C or 10 µM cRGDfV. The quantification is shown in (**C**) using SuperPlots, where each color represents a biological repeat (n=3) and the larger points represent the mean % Invasion for each repeat. (**D**) Quantification of spheres formed by AsPC-1 cells transfected with a control siRNA or P2Y_2_ siRNA and treated with or without 100 µM ATP. (**E**) Bright field and fluorescent images of spheres formed by AsPC-1 cells subjected to CRISPR/Cas9 gene disruption using a control guide RNA (CTR^CRISPR^) or P2Y_2_ guide RNAs (P2Y_2_^CRISPR^) and treated with or without 100 µM ATP. Quantification in (**F**). (**G, I**) Bright field and fluorescent images of AsPC-1 P2Y_2_^CRISPR^ cells or PANC-1 cells (respectively) transfected with wild-type *P2RY2* (P2Y_2_^RGD^) or mutant *P2RY2^D97E^* (P2Y_2_^RGE^) treated with or without 100 µM ATP and its quantification in (**H**) and (**J**), respectively. Statistical analysis with Kuskal-Wallis multiple comparison tests.

To evaluate the impact of P2Y_2_ in pancreatic cancer cell invasion, we used a 3D hanging drop spheroid model (Murray et al., 2022). PDAC cell lines were combined with stellate cells in a ratio of 1:2 (Kadaba et al., 2013), using an immortalized stellate cell line, PS-1 (Froeling et al., 2009) to form spheres (Figure 3A), recapitulating the ratios of the two biggest cellular components in PDAC. Stellate cells are crucial for successful hanging drop sphere formation (Figure 3—figure supplement 1A) and cancer cell invasion (Murray et al., 2022). Spheres were embedded in a Collagen type I and Matrigel mix and cultured for 48 hr until imaging and fixing (Figure 3A). Given that extracellular ATP concentration in tumors is in the hundred micromolar range (Pellegatti et al., 2008), spheres were treated with P2Y_2_ agonists ATP and UTP (100 µM). Both nucleotides increased invasion of the PDAC cell line AsPC-1 significantly compared to vehicle control (p<0.0001 and p*=*0.0013, respectively), and this was blocked by the P2Y_2_ selective antagonist AR-C (5 µM, p=0.0237, and p=0.0133; Figure 3B and C; Figure 3—figure supplement 1B). Treating spheres with AR-C on its own did not show significant effects on invasion (Figure 3—figure supplement 1B). Importantly, a non-hydrolyzable ATP (ATPγS;100 µM) showed similar effects to ATP, implicating ATP and not its metabolites as the cause of the invasion (Figure 3—figure supplement 1C). Of note, IF staining of PS-1 cells showed negligible expression of P2Y_2_ (Figure 3—figure supplement 1D). To determine whether integrin association was necessary for ATP-driven invasion, we treated spheres with 10 µM cyclic RGDfV peptide (cRGDfV), which binds predominantly to αVβ3 to block integrin binding to RGD motifs (Kapp et al., 2017), such as that in P2Y_2_ (Ibuka et al., 2015). cRGDfV treatment reduced ATP-driven motility significantly, both in 3D spheroid invasion assays (p<0.0001) (Figure 3B and C) and in 2D Incucyte migration assays (Figure 3—figure supplement 1E, F) as did treatment with AR-C. To ensure that this behavior was not restricted to AsPC-1 cells, experiments were corroborated in the epithelial-like BxPC-3 cell line (Figure 3—figure supplement 1G, H; Tan et al., 1986).

**Figure 3—figure supplement 1.**
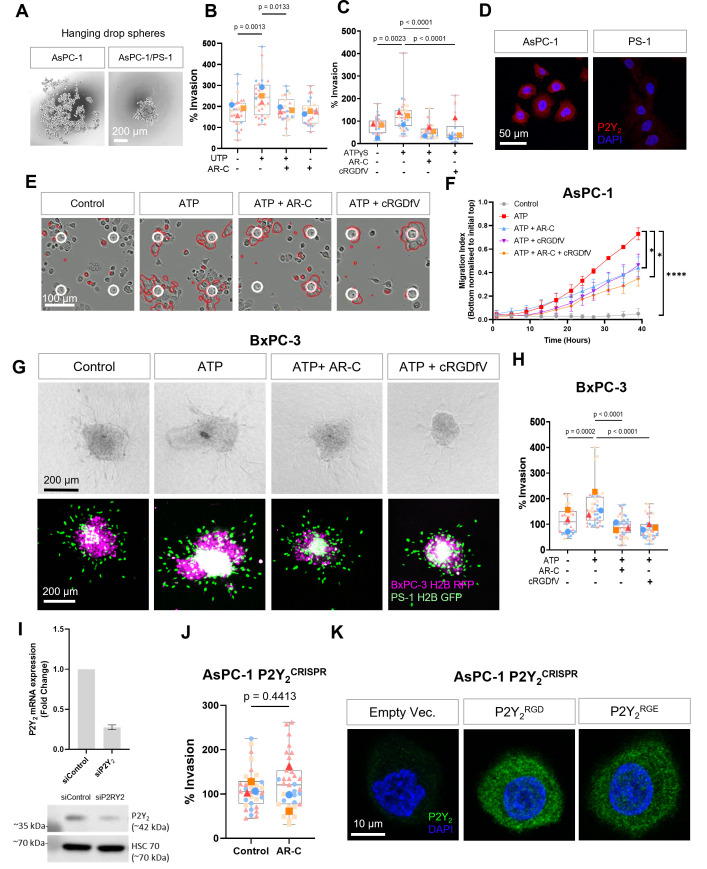
Invasion and migration experiments in Pancreatic ductal adenocarcinoma (PDAC) cell lines. (**A**) Hanging drop sphere with and without PS-1 cells. (**B, C**) Quantification of AsPC-1 spheres treated with 100 µM UTP or ATPγS (respectively) in the absence or together with 5 µM AR-C or 10 µM cRGDfV (n=3 biological replicates). (**D**) IF staining of P2Y_2_ in AsPC-1 and PS-1 stellate cells. (**E**) Migration assay with AsPC-1 and 100 µM ATP in the absence or together with 5 µM AR-C or/and 10 µM cRGDfV and (**F**) its quantification (n=3 biological replicates, statistical analysis with one-way ANOVA, * *p* < 0.5, **** *p* < 0.0001, error bars show standard deviation). (**G**) 3D sphere invasion assay using BxPC-3 cells treated with 100 µM of ATP in the absence or together with 5 µM AR-C or/and 10 µM cRGDfV and (**H**) its quantification (n=3 biological replicates). (**I**) qPCR of P2Y_2_ expression and western blot of siRNA treated cells (control siRNA and P2Y_2_ targeting siRNA, n=3 biological replicates). (**J**) AsPC-1 P2Y_2_^CRISPR^ spheres treated with or without 5 µM of AR-C (n=3 biological replicates). (**K**) P2Y_2_ IF staining of AsPC-1 P2Y_2_^CRISPR^ cells transfected with an empty vector, P2Y_2_^RGD^ or P2Y_2_^RGE^ plasmids. Statistical analysis of all invasion experiments with Kuskal-Wallis multiple comparison tests. Figure 3—figure supplement 1—source data 1.Labeled uncropped blot of Figure 3—figure supplement 1I. Figure 3—figure supplement 1—source data 2.Full unedited blot of Figure 3—figure supplement 1I.

To further verify that ATP-driven invasion was dependent on P2Y_2_, we silenced P2Y_2_ expression in AsPC-1 cells using siRNA (Figure 3D; Figure 3—figure supplement 1I), abrogating the invasive response to ATP (p<0.0001). P2Y_2_ involvement in this phenomenon was confirmed by generating a P2Y_2_ CRISPR-Cas9 AsPC-1 cell line (P2Y_2_^CRISPR^), which displayed a significant decrease in invasion compared to a control guide RNA CRISPR cell line (CTR^CRISPR^) in both ATP-treated (p<0.0001) and non-treated (p=0.0005) conditions (Figure 3F and E). Additionally, we tested the off-target effects of AR-C in AsPC-1 P2Y_2_^CRISPR^ spheres and confirmed no significant difference in invasion compared to the control (Figure 3—figure supplement 1J). Together, these findings demonstrate that P2Y_2_ is essential for ATP-driven cancer cell invasion.

To determine the importance of the RGD motif of P2Y_2_ in ATP-driven invasion, we obtained a mutant P2Y_2_ construct, where the RGD motif was replaced by RGE (P2Y_2_^RGE^), which has less affinity for αV integrins (Erb et al., 2001). This mutant was transfected into AsPC-1 P2Y_2_^CRISPR^ cells and compared to cells transfected with wild-type P2Y_2_ (P2Y_2_^RGD^; Figure 3—figure supplement 1K). Only spheres containing P2Y_2_^RGD^ transfected cells demonstrated a rescue of the ATP-driven invasive phenotype (p<0.0001; Figure 3G and H), with P2Y_2_^RGE^ spheres not responding to ATP treatment. To ensure this behavior was not influenced by off-target CRISPR effects, we repeated the experiment in PANC-1 cell line, which expresses very low levels of P2Y_2,_ but high levels of integrin αV (Figure 2—figure supplement 1F, G). No ATP-driven invasion was observed in PANC-1 cells transfected with an empty vector (EV) or with P2Y_2_^RGE^ (Figure 3I and J). Only when transfecting PANC-1 cells with P2Y_2_^RGD^ was ATP-driven invasion observed (p<0.0001). These results demonstrate that the RGD motif of P2Y_2_ is required for ATP-driven cancer cell invasion.

### DNA-PAINT reveals RGD-dependent changes in P2Y_2_ and integrin αV surface expression

**Figure 4.**
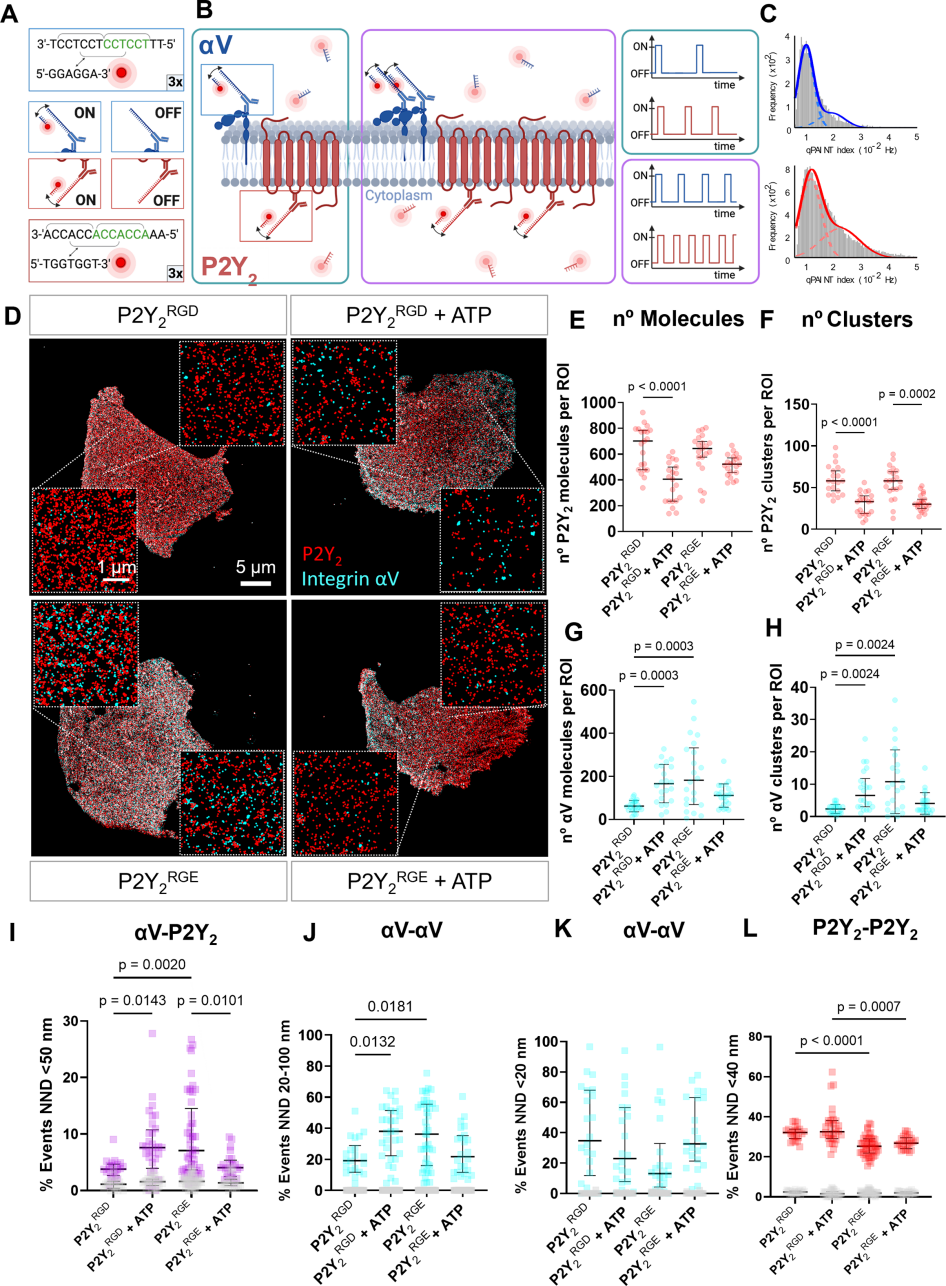
DNA-PAINT super-resolution microscopy reveals ATP and RGD-dependent changes in the number and distribution of integrin αV and P2Y_2_ molecules in the plasma membrane. (**A, B**) Overview of the DNA-PAINT microscopy technique and qPAINT analysis pipeline. (**C**) Histogram of qPAINT indices for αV (blue) and P2Y_2_ (red) single-molecule localization clusters. Solid lines represent multi-peak Gaussian fit. (**D**) Rendered DNA-PAINT images of AsPC-1 P2Y_2_^CRISPR^ cells transfected with P2Y_2_^RGD^ or P2Y_2_^RGE^ with or without 100 µM of ATP and close-ups showing the protein maps reconstructed from DNA-PAINT localization maps of P2Y_2_ (red) and integrin αV (cyan). The quantification of the number of proteins or protein clusters (>3 proteins) in each region of interest (ROI) are for P2Y_2_ (red) (**E**) and (**F**), respectively and integrin αV (cyan) (**G**) and (**H**), respectively. Quantification of protein proximity using the nearest neighbor distance (NND), with the percentages of integrin αV and P2Y_2_ proteins being <50 nm apart (**I**), between different αV integrins being 20–100 nm (**J**) or <20 nm (**K**) apart; and P2Y_2_ from other P2Y_2_ proteins being <40 nm apart (**H**). Statistical analysis with Kuskal-Wallis multiple comparison test of 21 4x4 µm ROIs from a minimum of 5 cell regions per condition.

To interrogate how P2Y_2_ interacts with αV integrins, we examined the nanoscale organization of P2Y_2_ and αV proteins under different treatment conditions using a multi-color quantitative super-resolution fluorescence imaging method, DNA-PAINT. DNA-PAINT is a single-molecule localization microscopy (SMLM) method based on the transient binding between two short single-stranded DNAs - the ‘imager’ and ‘docking’ strands. The imager strand is fluorescently labeled and freely diffusing in solution, whilst the docking strand is chemically coupled to antibodies targeting the protein of interest. For DNA-PAINT imaging of P2Y_2_ and integrin αV, proteins were labeled with primary antibodies chemically coupled to orthogonal docking sequences featuring a repetitive (ACC)n or (TCC)n motif, respectively (Figure 4A). The benefit of such sequences is to increase the frequency of binding events, which in turn allows the use of relatively low imager strand concentrations without compromising overall imaging times, whilst achieving a high signal-to-noise ratio and single-molecule localization precision (Strauss and Jungmann, 2020).

**Figure 4—figure supplement 1.**
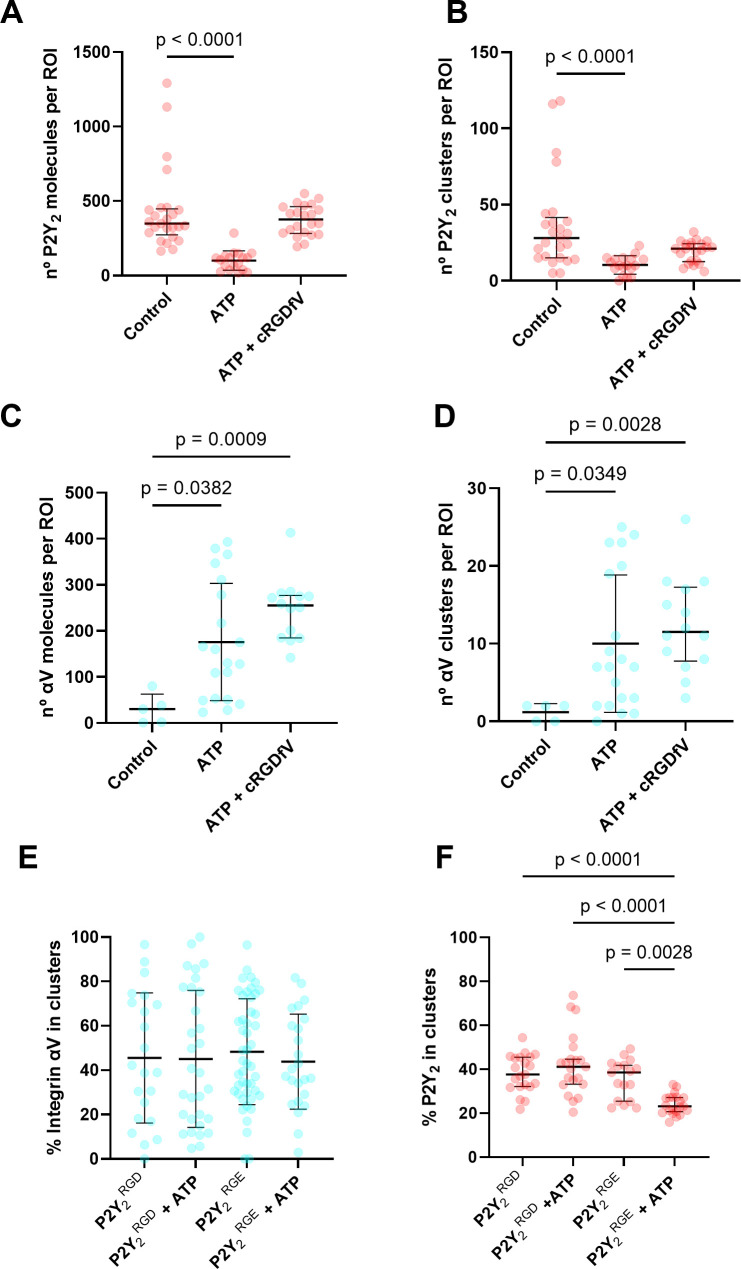
Quantification of P2Y2 and integrin αV at the membrane using DNA-PAINT.

**Figure 4—figure supplement 2.**
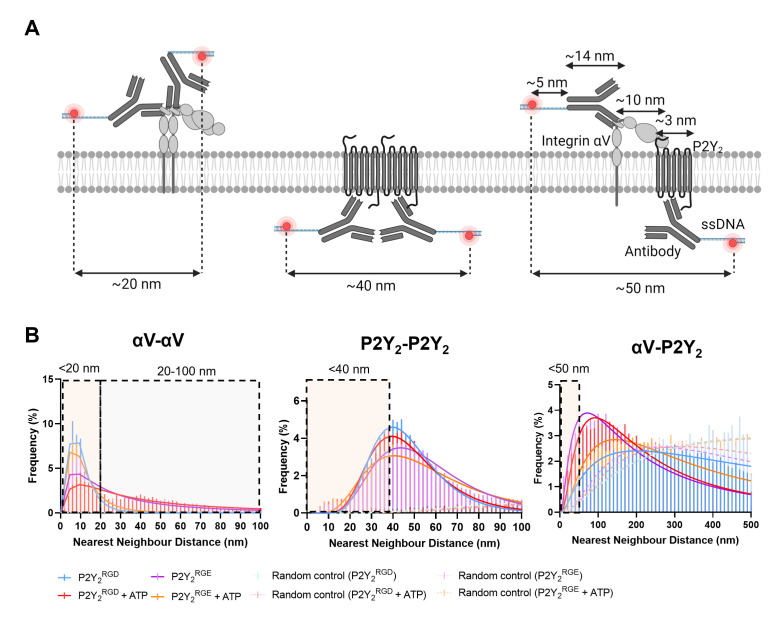
Schematic diagram of nearest neighbor distance (NND) distances and NND histograms. (**A**) Schematic diagram of the predicted maximum distance between fluorescent molecules indicating physical contact between proteins, to the nearest first significant figure. (**B**) Histograms of the nearest neighbor distance between proteins vs the frequency of occurrence for AsPC-1 P2Y_2_^CRISPR^ in different conditions (solid line, strong color) or randomly computer-generated controls (dotted line, light color).

The repetitive binding of imager and docking DNA strands in DNA-PAINT causes the same protein to be detected multiple times with nearly identical coordinates, originating a cluster of single-molecule localization around the true position of the protein. In contrast to other SMLM approaches, it is possible to take advantage of the DNA-binding kinetics to stoichiometrically calculate the number of proteins detected in each cluster of single molecule localizations, via an approach known as qPAINT (Schnitzbauer et al., 2017). As exemplified in Figure 4B (and detailed in the methods section), qPAINT relies on the first-order binding kinetics between the individual imager and docking strands to determine the number of copies of a protein that reside within a cluster of single-molecule localizations. The qPAINT index histograms obtained from P2Y_2_ and αV DNA-PAINT data sets were fitted with a multi-peak Gaussian function, identifying peaks located at multiples of a qPAINT index value of $q_{i,1}$ 0.011 Hz and 0.009 for the P2Y_2_ and αV docking-imager pairs, respectively (Figure 4C). These values were thus used to quantify the exact number of P2Y_2_ and αV proteins in all the clusters of single-molecule localization in the DNA-PAINT data sets. By combining qPAINT with spatial statistics, we recovered a good estimation of the ground truth position of all the proteins in the DNA-PAINT data and quantified protein clustering.

We have previously analyzed GPCR oligomerization quantitatively using DNA-PAINT super-resolution microscopy of P2Y_2_ in AsPC-1 cells (Joseph et al., 2021), where we observed a decrease in P2Y_2_ oligomerization upon AR-C treatment. Hence, we questioned whether the RGD motif in P2Y_2_ affected receptor distribution and clustering. We imaged AsPC-1 P2Y_2_^CRISPR^ cells transfected with P2Y_2_^RGD^ or P2Y_2_^RGE^ in the absence or presence of 100 µM ATP for 1 hr (Figure 4D), observing a 42% decrease in the median density of P2Y_2_ proteins at the membrane when P2Y_2_^RGD^ cells were treated with ATP, compared to control (p<0.0001; Figure 4E). In contrast, although a slight decrease in the density of P2Y_2_ proteins on P2Y_2_^RGE^ cells was observed following ATP treatment, this was not statistically significant (p=0.1570). The density of P2Y_2_ proteins and protein clusters in both P2Y_2_^RGD^ and P2Y_2_^RGE^ controls were equivalent (Figure 4E and F; p>0.9999), indicating similar expression of the receptor at the surface in both control conditions. Interestingly, the density of P2Y_2_ clusters decreased significantly in both conditions when treated with ATP (Figure 4F; 43% decrease, p<0.0001 for P2Y_2_^RGD^, and 48% decrease, p=0.0002 for P2Y_2_^RGE^). We repeated these studies with normal AsPC-1 cells (untransfected and with unaltered P2Y_2_ expression) treated with ATP +/-cRGDfV, only observing a reduction of P2Y_2_ at the membrane with ATP alone (68% decrease, p<0.0001), while co-treatment with cRGDfV prevented this change (p>0.9999; Figure 4—figure supplement 1A, B). These findings highlight that the RGD motif is required for αV integrin to control P2Y_2_ levels at the membrane.

Turning to αV integrins, we observed an increase in the density of αV molecules and αV clusters at the membrane when stimulating P2Y_2_^RGD^ with ATP (165 αV molecules/ROI, IQR = 162.75; 6.5 αV clusters/ROI, IQR = 8.75) compared to P2Y_2_^RGD^ without stimulation (58 αV molecules/ROI, IQR = 41; 2.5 αV clusters/ROI, IQR = 2; p=0.0003; Figure 4G and H). This phenomenon was also observed with normal AsPC-1 cells, with significantly more αV molecules and clusters (p=0.0382 and p=0.0349) detected following ATP stimulation (Figure 4—figure supplement 1C, D). In absence of stimulation, P2Y_2_^RGE^ transfected cells exhibited more αV molecules and clusters at the membrane (182 αV molecules/ROI, IQR = 262.75; 9 αV clusters/ROI IQR = 14) compared to P2Y_2_^RGD^ cells (p=0.0003, p=0.0024, respectively). However, treating P2Y_2_^RGE^ cells with ATP did not result in significant changes in αV molecules and clusters (p=0.7086; p=0.1846). When the number of clusters was normalized with the number of αV molecules, to obtain the percentage of αV in clusters (Figure 4—figure supplement 1E), there was no significant difference between conditions (p>0.9999), indicating that the increase in the number of αV clusters was due to an increase in the number of αV proteins at the membrane. Of note, the percentage of P2Y_2_ clusters significantly decreased in P2Y_2_^RGE^ cells when treated with ATP compared to all other conditions (Figure 4—figure supplement 1F). Taken together, these data indicate an RGD motif-dependent function of activated P2Y_2_ in localizing integrin αV to the membrane.

Nearest neighbor distance (NND) was used to analyze homo and heterotypic protein-protein interactions between P2Y_2_ and αV. NND ranges were selected by using the approximate dimension of the antibodies (~14 nm) (Tan, 2008), integrins (5–10 nm) (Lepzelter et al., 2012), and GPCRs (~3 nm) (Figure 4—figure supplement 2A) and corroborating them with the NND histograms (Figure 4—figure supplement 2B) to predict the NND range in nm indicating a protein-protein interaction. We detected a higher percentage of integrin αV proteins in <50 nm proximity to P2Y_2_ in P2Y_2_^RGD^ cells following ATP stimulation (Figure 4I; 103% increase, p=0.0143). In contrast, P2Y_2_^RGE^ cells stimulated with ATP showed a 43% decrease (p=0.0101) in αV molecules in close proximity to P2Y_2_ in comparison to unstimulated cells. Analyzing the percentage of αV proteins with NND in the 20–100 nm range, we saw a similar pattern (Figure 4J). ATP-stimulated P2Y_2_^RGD^ and unstimulated P2Y_2_^RGE^ cells showed an increased percentage of αV proteins spaced at this range compared to untreated P2Y_2_^RGD^ cells (98% increase with p=0.0132 and 89% increase with p=0.0181). No significant changes were observed in NND of <20 nm between αV proteins in any of the conditions (Figure 4K). In contrast, P2Y_2_^RGD^ molecules were in significantly closer proximity to each other compared to P2Y_2_^RGE^ in control and stimulated conditions (p<0.0001 and p=0.007) (Figure 4L). In summary, our SMLM studies demonstrate a reciprocal interaction between αV integrin and P2Y_2_ receptors, where P2Y_2_ can alter integrin localization to the plasma membrane while αV integrins influence activated P2Y_2_ membrane localization.

### The RGD motif in P2Y_2_ is involved in integrin signaling

**Figure 5.**
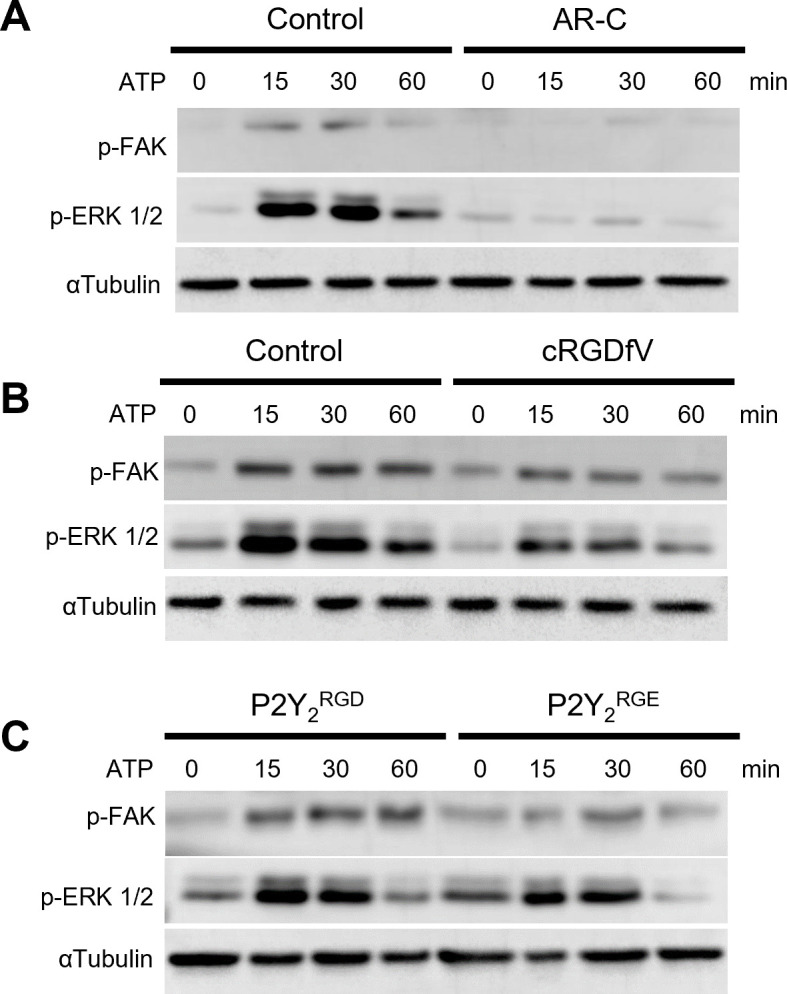
The RGD motif in P2Y_2_ is involved in FAK/ERK signaling. (**A, B**) Western blots of phosphorylated FAK (p-FAK) and ERK (p-ERK) of AsPC-1 cells treated with ATP or pre-treated for 30 min with AR-C (5 µM) or cRGDfV (10 µM), respectively and treated with ATP for 60 min. (**C**) Western blot of AsPC-1 P2Y_2_^CRISPR^ cells transfected with P2Y_2_^RGD^ or P2Y_2_^RGE^ and treated with ATP for 60 min. Representative images of three biological replicates. Figure 5—source data 1.Labeled uncropped blots of Figure 5. Figure 5—source data 2.Full unedited blots of Figure 5.

There is growing evidence of the importance of endosomal GPCR signaling and its potential relevance in disease and therapeutic opportunities (Calebiro and Godbole, 2018). As we identified the RGD motif in P2Y_2_ having a possible role in receptor internalization, integrin dynamics, and invasion, we proceeded to look at integrin signaling through phosphorylation of FAK (p-FAK) and ERK (p-ERK) from 0 to 1 hr after treating with 100 µM ATP. AsPC-1 cells displayed a significant increase of FAK and ERK phosphorylation after 15 min of ATP stimulation, which was abrogated by concomitant targeting of P2Y_2_ with AR-C (Figure 5A). When impairing the RGD motif function in P2Y_2_ with cRGDfV or by transfecting AsPC-1 P2Y_2_^CRISPR^ cells with the P2Y_2_^RGE^ mutant, p-FAK, and p-ERK levels decreased (Figure 5B and C). Collectively, targeting the RGD motif in P2Y_2_ impairs receptor signaling and inhibits pancreatic cancer cell invasion.

## Discussion

Improved molecular understanding of PDAC is vital to identify effective therapeutic approaches to improve patient survival. Purinergic signaling includes many druggable targets that have been related to hypoxia (Synnestvedt et al., 2002), immunosuppression (Fong et al., 2020), and invasion (Li et al., 2015), but have been relatively underexplored in PDAC. In this study, we used publicly available databases to identify purinergic signaling genes that could be promising targets for PDAC, determining P2Y_2_ as a driver of pancreatic cancer cell invasion. Extracellular ATP stimulated invasion in a 3D spheroid model of PDAC; an effect blocked by targeting P2Y_2_ genetically and pharmacologically. Mechanistically, we identified that the RGD motif in the first extracellular loop of P2Y_2_ is required for ATP-driven cancer invasion. Importantly, quantitative DNA-PAINT super-resolution fluorescence microscopy revealed the role of this RGD motif in orchestrating the number of P2Y_2_ and αV integrin proteins at the plasma membrane, upon ATP stimulation.

Purinergic signaling has been associated classically with hypoxia and immune function in cancer (Di Virgilio et al., 2018). One of the first reports of hypoxia-inducing ATP release in cells identified an increase of extracellular ATP in rat heart cells when kept in hypoxic conditions (Forrester and Williams, 1977). PDAC is a highly hypoxic cancer, with high levels of ATP reported in the tumor interstitial fluid of human and mouse PDAC tissues compared to healthy tissues (Hu et al., 2019). This vast release of ATP results in immune-mediated inflammatory responses via immune cells expressing purinergic signaling receptors (Chiarella et al., 2021). Expression of most purinergic genes was associated predominantly with immune cells, immunogenic PDAC subtype, and low hypoxia scores (Figure 1C and E). In contrast, expression of genes correlated with worse survival and hypoxia (*PANX1*, *NT5E*, *ADORA2B,* and *P2RY2*) was associated with tumor cells and the squamous PDAC subtype, correlating with hypoxia, inflammation, and worse prognosis (Bailey et al., 2016). The role of CD73 in PDAC has been examined in several studies (Yu et al., 2021) (NCT03454451, NCT03454451). In contrast, the adenosine A_2B_ receptor has not been well studied. Adenosine A_2B_ receptor requires larger agonist concentrations for activation compared to other receptors in the same family, such as adenosine A_2A_ (Bruns et al., 1986; Xing et al., 2016), and receptor expression has been reported to increase when cells are subjected to hypoxia (Feoktistov et al., 2004). Moreover, HIF-1α has been shown to upregulate A_2B_ and P2Y_2_ expression in liver cancer (Tak et al., 2016; Kwon et al., 2019). From our analyses, P2Y_2_ was associated with the worst patient overall survival, highest patient hypoxia scores, and strongest correlation with cancer cell expression compared to other purinergic genes. These observations were supported by published immunohistochemical staining of 264 human PDAC samples, showing that P2Y_2_ localized predominantly in cancer cells in human PDAC and that P2Y_2_ activation with ATP led to elevated HIF-1α expression (Hu et al., 2019). Hence, we decided here to explore P2Y_2_ in greater depth.

P2Y_2_ has been associated with cancer cell growth and glycolysis in PDAC (Ko et al., 2012; Hu et al., 2019; Wang et al., 2020). Combination treatment of subcutaneous xenografts of AsPC-1 or BxPC-3 cells with the P2Y_2_ antagonist AR-C together with gemcitabine significantly decreased tumor weight and resulted in increased survival compared to placebo or gemcitabine monotherapy control (Hu et al., 2019). Surprisingly, GSEA results of two different cohorts suggested a possible additional function of P2Y_2_ in invasion. Increased glycolysis and cytoskeletal rearrangements have been linked (Park et al., 2020), and both events could occur downstream of P2Y_2_ activation. P2Y_2_ has been implicated in invasive phenotypes in prostate, breast, and ovarian cancer (Jin et al., 2014; Li et al., 2015; Martínez-Ramírez et al., 2016). Moreover, high P2Y_2_ expression in patients was related to integrin signaling. The RGD motif in the first extracellular loop of P2Y_2_ results in a direct interaction of P2Y_2_ with RGD-binding integrins, particularly integrins αVβ3 and αVβ5 (Erb et al., 2001; Ibuka et al., 2015). This interaction can exert phenotypic effects – for example, the binding of P2Y_2_ to integrins via its RGD motif is necessary for tubule formation in epithelial intestinal cell line 3D models (Ibuka et al., 2015). We focus here on the importance of the RGD motif of P2Y_2_ and its key for integrin interaction in a cancer context. We were able to abrogate ATP-driven invasion using either the P2Y_2_ selective antagonist AR-C or by blocking P2Y_2_-integrin complexes using the selective αVβ3 cyclic RGD-mimetic peptide inhibitor cRGDfV. Likewise, spheres made using ASPC-1 P2Y_2_^CRISPR^ or PANC-1 cells transfected with mutant P2Y_2_^RGE^, which decreases the affinity of P2Y_2_ for integrins, did not invade in response to ATP stimulation. Altogether, these results (1) support P2Y_2_ involvement in PDAC cell invasion, (2) show the RGD motif is essential for this function, and (3) identify the mechanism for this to be caused by P2Y_2_-integrin complexes. Despite efforts, there are currently no clinically efficacious P2Y_2_ antagonists, with poor oral bioavailability and low selectivity being major issues (Neumann et al., 2022). Our findings demonstrate that P2Y_2_ can also be targeted by blocking its interaction with RGD-binding integrins, due to its dependence on integrins for its pro-invasive function.

GPCR-integrin crosstalk is involved in many biological processes (Wang et al., 2005; Teoh et al., 2012). Only one study has directly examined the spatial distribution of integrins and GPCRs, however, this relied on IF analysis (Erb et al., 2001), where only changes in the micron scale will be perceived, hence losing information on the nanoscale distances and individual protein interactions. Here, we present a method to image integrin and GPCR dynamics using quantitative DNA-PAINT super-resolution fluorescence microscopy (Schnitzbauer et al., 2017), allowing spatial and quantitative assessment of P2Y_2_ and integrin αV interactions at the single protein level. Following ATP stimulation, the number of P2Y_2_ proteins at the plasma membrane decreased significantly after 1 hr, implying receptor internalization, in line with previous work showing P2Y_2_ at the cell surface was reduced significantly after 1 hr of UTP stimulation (Tulapurkar et al., 2005). Of note, cytoskeletal rearrangements, which we have also observed upon ATP stimulation (Figure 2E), were required for P2Y_2_ clathrin-mediated internalization, and authors noted that P2Y_2_ was most likely in a complex with integrins and extracellular matrix-binding proteins. Cells expressing RGE mutant P2Y_2_ or treated with cRGDfV, did not show significant changes in P2Y_2_ levels at the membrane upon ATP treatment, thus implicating the RGD motif in P2Y_2_ in agonist-dependent receptor internalization, though we have focused on motility phenotype in this work.

**Figure 6.**
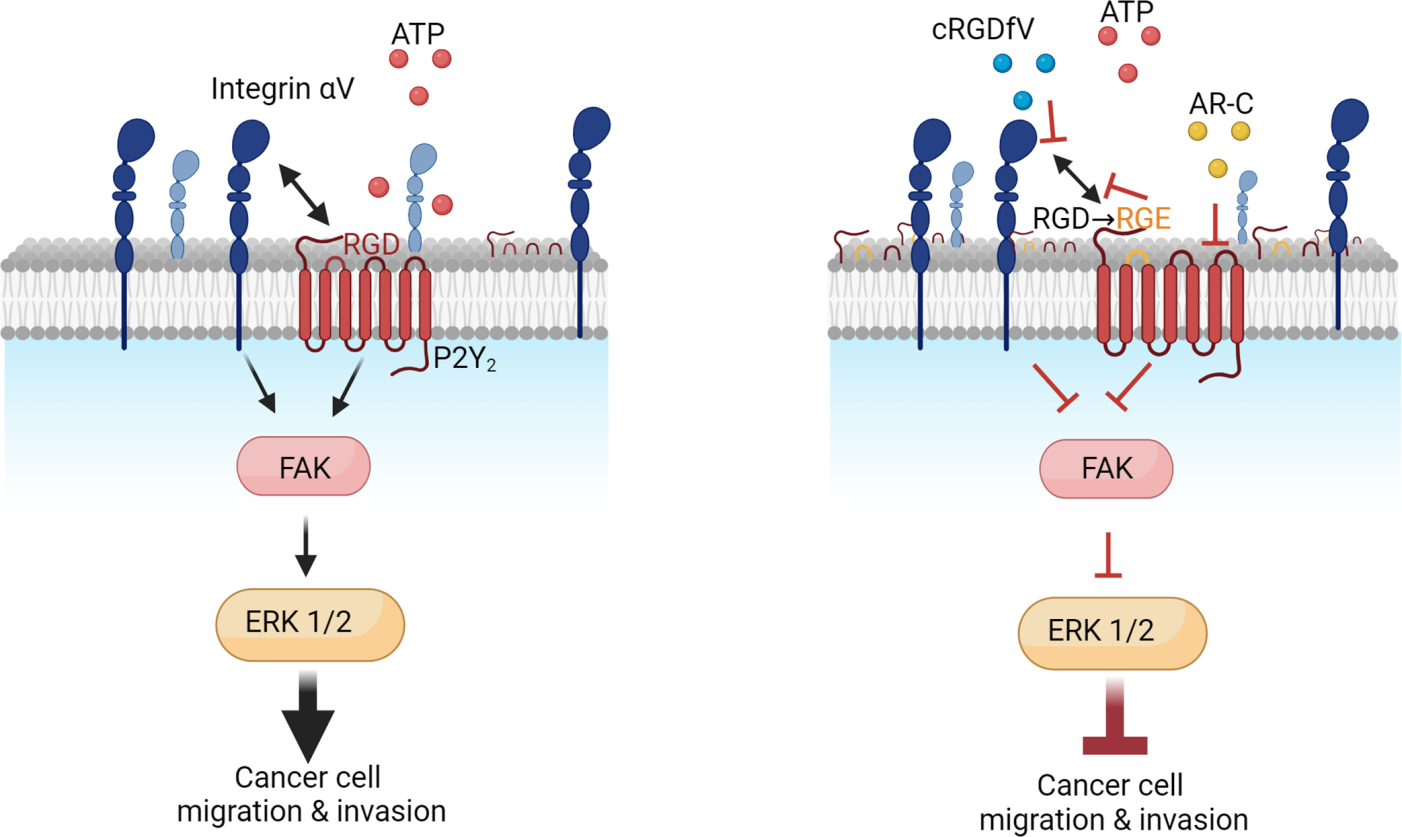
Proposed mechanism of P2Y_2_ and integrin interactions in pancreatic cancer invasion.

P2Y_2_ affecting cell surface redistribution of αV integrin has been reported, with αV integrin clusters observed after 5 min stimulation with UTP (Chorna et al., 2007). We observed an increased number of αV integrin molecules and clusters 1 hr after ATP stimulation, although this increase in clusters was mainly due to the increase in the total number of αV integrins at the membrane. The distance between αV integrin and P2Y_2_ molecules decreased (NND <50 nm) with ATP stimulation, indicating possible interaction. In contrast, with mutant P2Y_2_^RGE^, no significant ATP-dependent changes in the number of P2Y_2_ or αV integrin proteins at the membrane were observed. The same phenomenon was observed when treating normal AsPC-1 cells (untransfected and with no alteration to P2Y_2_) with cRGDfV and ATP. We speculate that by reducing the ability of integrins to bind to the RGD of P2Y_2_, through receptor internalization, RGE mutation or through cRGDfV treatment, there is less RGD-triggered integrin endocytosis, hence less integrin recycling and an increase of integrins at the cell surface. Western blot results supported our postulated role of the RGD motif in P2Y_2_ regulating downstream integrin signaling through FAK and ERK, leading to cancer cell migration and invasion (Figures 5 and 6). This is the first single-molecule super-resolution study to explore integrin and GPCR dynamics and to demonstrate a requirement for integrin-P2Y_2_ interactions in cancer cell invasion.

In summary, our study demonstrates that P2Y_2_, via its RGD motif, has a pivotal role in ATP-induced PDAC invasion by interacting with and regulating the number of αV integrins at the plasma membrane, revealing this critical axis as a promising therapeutic target.

## Methods

### Data mining and bioinformatic analysis

Hazard ratios and the P2Y_2_ Kaplan-Meier plot for overall survival were obtained using Kaplan-Meier Plotter (RRID:SCR_018753) (Lánczky and Győrffy, 2021) and the pancreatic adenocarcinoma dataset from the cancer genome atlas (PAAD TCGA, RRID:SCR_003193).

Using cBioPortal (RRID:SCR_014555) (Gao, 2013) and the database PAAD TCGA, mRNA differential expression analysis was performed for each Hypoxia Score (Winter et al., 2007; Buffa et al., 2010; Ragnum et al., 2015) by separating patients using the median hypoxia score. Results from purinergic genes were plotted in a volcano plot using VolcaNoseR (Goedhart and Luijsterburg, 2020). Significant hits were plotted in a heat map using cBioPortal (Gao, 2013). RNAseq raw counts from stromal and epithelial PDAC tissue from microdissections were downloaded from the GEO database (GSE93326) (Maurer et al., 2019) and a differential expression analysis was performed using DESeq2 (RRID:SCR_015687) (Love et al., 2014; Varet et al., 2016) in R.

Gene weight results from DECODER from PDAC tissues in the TCGA database were obtained from published results (Peng et al., 2019). Using GEPIA (RRID:SCR_018294) (Tang et al., 2017), mRNA expression of purinergic genes in normal tissue from the Genotype-Tissue Expression (GTEx, RRID:SCR_013042) compared to cancer tissue (PAAD TCGA) was obtained. PDAC cell line mRNA z-scores or mRNA reads per kilobase million (RPKM) were obtained using cBioPortal and the Cancer Cell Line Encyclopaedia (CCLE, RRID:SCR_013836) data (Gao, 2013).

For gene set enrichment analysis (GSEA), cBioPortal was used to separate PAAD TCGA or PDAC CPTAC patients into high and low *P2RY2* by *P2RY2* median expression and perform the differential expression analysis. Log ratio values were inserted in the WEB-based Gene SeT AnaLysis Toolkit (WebGestalt, RRID:SCR_006786) (Liao et al., 2019), where ‘GO: Molecular Function’ or ‘Panther’ with default analysis parameters were selected.

### RNAscope in-situ hybridization

Formalin-fixed paraffin-mbedded (FFPE) sections (n=3) of PDAC with stroma and normal adjacent tissue were obtained from the Barts Pancreas Tissue Bank (Project 2021/02/QM/RG/E/FFPE). Sections were stained using the human *P2RY2* probe (853761, ACD) and the RNAscope 2.5 HD Assay-RED (ACD) following the manufacturer’s instructions. Slides were imaged by NanoZoomer S210 slide scanner (Hamamatsu).

### Cell lines and cell culture

The pancreatic cancer cell lines AsPC-1 (RRID:CVCL_0152), BxPC-3 (RRID:CVCL_0186), MIA PaCa-2 (RRID:CVCL_0428) and PANC-1 (RRID:CVCL_0480), in addition to the immortalized stellate cell line PS-1 (Froeling et al., 2009) were kindly donated by Prof. Hemant Kocher (Queen Mary University of London). Cell lines stably expressing fluorescently labeled histone subunits (H2B) or Lifeact (Riedl et al., 2008) were transduced with viral supernatant obtained from HEK293T cells co-transfected with pCMVR8.2 (Addgene #12263) and pMD2.G (Addgene #12259) packaging plasmids, and either H2B-GFP (Addgene #11680), H2B-RFP (Addgene #26001), or Lifeact-EGFP (Addgene # 84383) plasmids using FuGENE transfection reagent (Promega), following manufacturer’s guidelines. Successfully transduced cells were isolated using a BD FACS Aria Fusion cell sorter. AsPC-1 P2Y_2_^CRISPR^ cells were generated by transfecting cells with a dual gRNA (TGAAGGGCCAGTGGTCGCCGCGG and CATCAGCGTGCACCGGTGTCTGG)CRISPR-CAS9 plasmid (VectorBuilder) with an mCherry marker which was used to select successfully transfected cells as above. Clonal expansion of single sorted cells was achieved with serial dilution cloning. Clones were evaluated by IF for P2Y_2_ compared to parental AsPC-1 cells. Cell lines were grown at 37 °C with 5% CO_2_ in DMEM (Gibco), RPMI-1640 (Gibco), or DMEM/F-12 (Sigma) supplemented with 10% fetal bovine serum (Sigma). Cells were monitored for mycoplasma contamination every six months.

### Cell fixation and immunofluorescent staining

Cells were seeded on coverslips placed in a six well-plate (Corning) and fixed the next day in 4% paraformaldehyde (LifeTech) for 30 min and washed 3 x with phosphate-buffered saline (PBS). Coverslips were placed in 0.1% Triton X-100 (Avantor) for 10 min for permeabilization, followed by three PBS washes and blocking with 5% bovine serum albumin (BSA; Merck) for 1 hr. Coverslips were incubated at 4 °C overnight with anti-P2Y_2_ (APR-010, Alomone labs) and anti-integrin αV antibodies (P2W7, Santa Cruz) diluted in blocking solution (1:100 and 1:200, respectively). After three PBS washes, coverslips were incubated for 1 hr with Alexa Fluor 647 goat anti-mouse and Alexa Fluor 488 goat anti-rabbit (Invitrogen) or Alexa Fluor 546 goat anti-rabbit at 1:1000, diluted in blocking buffer. Following three PBS washes, 4’,6-diamidino-2-phenylindole (DAPI, Sigma-Aldrich) was used as a nuclear stain and was incubated for 10 min. Slides were mounted using Mowiol (Calbiochem) and imaged 24 hr later using a LSM 710 confocal microscope (Zeiss).

### siRNA and plasmid transfection

Cells were seeded in six well plates at a density of 200,000 cells/well 24 hr before transfection. For siRNA experiments, cells were transfected with 20 nM pooled control or P2Y_2_-targeting siRNAs from a siGENOME SMARTpool (Dharmacon, GE Healthcare) with Lipofectamine 3000 (Invitrogen) following the manufacturer’s instructions. For P2Y_2_ plasmid expression experiments, cells were transfected with 500 nM *P2RY2* (P2Y_2_^RGD^) or *P2RY2D97E* (P2Y_2_^RGE^) in pcDNA3.1 vector (Obtained from GenScript) or pcDNA3.1 alone (Empty vector, EV) together with lipofectamine 3000 and p3000 reagent (Invitrogen) as per manufacturer’s instructions. Plasmid concentration was selected by comparing AsPC-1 IF staining of P2Y_2_ with IF staining in AsPC-1 P2Y_2_^CRISPR^ and PANC-1 cells with different concentrations of the plasmid to achieve a similar IF signal. Cells were split 48 hr post-transfection for experiments or imaged 72 hr post-transfection.

### 3D sphere model invasion assay

Spheres of PDAC cell lines with PS-1 cells were generated as described (Murray et al., 2022). Cancer cells at 22,000 cells/mL and PS-1 cells at 44,000 cells/mL were combined with DMEM/F-12 and 1.2% methylcellulose in a 4:1 ratio of methylcellulose (Sigma-Aldrich) and 20 µl drops, each containing 1000 cells, pipetted on the underside of a 15 cm dish lid (Corning) and hanging drops were incubated overnight at 37 °C. The next day, spheres were collected and centrifuged at 300 g for 4 min and washed with the medium. A mix of 2 mg/mL collagen (Corning), 175 µL/mL Matrigel, 25 µL/mL HEPES (1 M, pH 7.5), and 1 N NaOH (for neutral pH correction) was prepared with DMEM/F12 medium. Spheroids were re-suspended and seeded in low attachment 96-well plates (50 µl per well) with 40 µL previously gelled mix in the bottom of the wells. Once set, 150 µL of DMEM/F12 was added with treatments. Spheres were treated with 100 µM adenosine 5’-triphosphate trisodium salt hydrate (ATP, Sigma), uridine 5’-triphosphate trisodium salt hydrate (UTP, Sigma) or adenosine 5’-[γ-thio]triphosphate tetralithium salt (ATPγS, Tocris) alone or with 5 µM AR-C118925XX (AR-C, Tocris), or 10 µM cyclo(RGDfV) (cRGDfV, Sigma-Aldrich). Treatments were repeated 24 hr later. Spheres were imaged with a Zeiss Axiovert 135 light microscope at 10 x on day two after seeding. Cells were stained with 4’,6-diamidino-2-fenilindol (DAPI) (1:1000) for 10 min and imaged with a Zeiss LSM 710 confocal microscope. % Invasion was calculated by drawing an outline around the total area $A_{total}$ and central area $A_{central}$ of the spheres with ImageJ (Fiji) and using the equation:

$$\%Invasion=\;\left(\frac{A_{total}-A_{central}}{A_{central}\;}\right)x100$$

Results were plotted in SuperPlots by assigning different colors to repeats and superimposing a graph of the average % Invasion with a darker shade of the assigned color as described previously (Lord et al., 2020).

### IncuCyte migration assay

In IncuCyte ClearView 96-well cell migration plates (Essen BioScience), 40 μL medium with 5000 cells were seeded in each well. A solution of 20 μL medium with 15 µM AR-C or 30 µM cRGDfV was added on top of the wells to achieve a final concentration of 5 µM and 10 µM, respectively. Cells were allowed to settle for 15 min at room temperature and then placed at 37 °C for pre-incubation with the treatments for another 15 min. A volume of 200 μL of medium with or without 100 µM ATP was added in the appropriate reservoir wells and the plate was placed in the IncuCyte S3 (Essen BioScience) and was monitored every 4 hr for 39 hr (average doubling time of AsPC-1 cells Chen et al., 1982). Using the IncuCyte S3 2019 A software, the migration index was calculated by analyzing the average area occupied by the cells in the bottom well and was averaged with the initial average area occupied by cells in the top well.

### RNA extraction and qPCR analysis

RNA was extracted using the Monarch RNA extraction kit (New England BioLabs) as instructed by the manufacturer. The extracted RNA was quantified using a Nanodrop One Spectrophotometer (ThermoFisher Scientific). Using LunaScript RT Supermix kit (BioLabs), cDNA was prepared in a 20 μL reaction according to the manufacturer’s instructions. The resulting cDNA was used in conjunction with MegaMix-Blue and *P2RY2* primers (Eurogentec; Forward sequence: GCTACAGGTGCCGCTTCAAC, reverse sequence: AGACACAGCCAGGTGGAACAT) (Hu et al., 2019) for quantitative polymerase chain reaction (qPCR) at the manufacturer’s recommended settings in a StepOnePlus Real-Time PCR System (Applied Biosystems). The relative mRNA expression was calculated using the $2^{-∆∆Ct}$ method (Livak and Schmittgen, 2001) and normalized to GAPDH.

### DNA-antibody coupling reaction

DNA labeling of anti-αV antibody (P2W7, Santa Cruz, RRID:AB_627116) and anti-P2Y_2_ receptor antibody (APR-010, Alomone labs, RRID:AB_2040078) was performed via maleimidePEG2-succinimidyl ester coupling reaction as previously described (Simoncelli et al., 2020; Joseph et al., 2021). First, 30 µL of 250 mM DDT (Thermo Fisher Scientific) was added to 13 µL of 1 mM thiolated DNA sequences 5′-Thiol-AAACCACCACCACCA-3′ (Docking 1), and 5-Thiol-TTTCCTCCTCCTCCT-3’ (Docking 2) (Eurofins). The reduction reaction occurred under shaking conditions for 2 hrs. 30 min after the reduction of the thiol-DNA started, 175 µL of 0.8 mg/mL antibody solutions were incubated with 0.9 µL of 23.5 mM maleimide-PEG2-succinimidyl ester cross-linker solution (Sigma-Aldrich) on a shaker for 90 min at 4 °C in the dark. Prior to DNA-antibody conjugation, both sets of reactions were purified using Microspin Illustra G-25 columns (GE Healthcare) and Zeba spin desalting columns (7 K MWCO, Thermo Fisher Scientific), respectively, to remove excess reactants. Next, coupling of anti-P2Y_2_ with DNA docking 1 and anti-αV with DNA Docking 2 was performed by mixing the respective flow-through of the columns and incubate them overnight, in the dark, at 4 °C under shaking. Excess DNA was removed via Amicon spin filtration (100 K, Merck) and antibody-DNA concentration was measured using a NanoDrop One spectrophotometer (Thermo Fisher Scientific) and adjusted to 10 µM with PBS. Likewise, spectrophotometric analysis was performed to quantify the DNA-antibody coupling ratio and found to be ∼1.2 on average for both the oligo-coupled primary antibodies.

### Cell fixation and immunofluorescence staining for DNA-PAINT imaging

Cells were seeded at 30,000 cells per channel on a six-channel glass-bottomed microscopy chamber (μ-SlideVI^0.5^, Ibidi) pre-coated with rat tail collagen type I (Corning). The chamber was incubated at 37 °C for 8 hr before treatments. Cells were treated with 100 μM of ATP (or the equivalent volume of PBS as control) in the medium for 1 hr and were fixed and permeabilized as described in the ‘Cell fixation and immunofluorescent staining’ section. Following permeabilization, samples were treated with 50 mM ammonium chloride solution (Avantor) for 5–10 min to quench auto-fluorescence and cells were washed 3x in PBS. Blocking was completed via incubation with 5% BSA (Merck) solution for 1 hr followed by overnight incubation at 4 °C with 1:100 dilutions of DNA labeled anti-P2Y_2_, and DNA labeled anti-αV antibody in blocking solution. The next day, samples were washed 3× in PBS and 150 nm gold nanoparticles (Sigma-Aldrich) were added for 15 min to act as fiducial markers for drift correction, excess of nanoparticles were removed by 3x washes with PBS. Samples were then left in DNA-PAINT imager buffer solution, prepared as described below, and immediately used for DNA-PAINT imaging experiments.

### DNA-PAINT imager solutions

A 0.1 nM P2Y_2_ imager strand buffer solution (5-TTGTGGT-3’-Atto643, Eurofins) and a 0.2 nM αV imager strand buffer solution (5-GGAGGA-3’-Atto643, Eurofins) were made using 1x PCA (Sigma-Aldrich), 1x PCD (Sigma-Aldrich), 1x Trolox (Sigma-Aldrich), 1x PBS, and 500  mM NaCl (Merck) which facilitates the establishment of an oxygen scavenging and triplet state quencher system. Solutions were incubated for 1 hr in the dark before use. Stock solutions of PCA, PCD, and Trolox were prepared as follows: 40x PCA (protocatechuic acid) stock was made from 154 mg of PCA (Sigma-Aldrich) in 10 mL of Ultrapure Distilled water (Invitrogen) adjusted to pH 9.0 with NaOH (Avantor, Radnor Township, PA, USA). 100 x PCD (protocatechuate 3,4-dioxygenase) solution was made by adding 2.2 mg of PCD (Sigma-Aldrich) to 3.4 mL of 50% glycerol (Sigma-Aldrich) with 50 mM KCl (Sigma-Aldrich), 1 mM EDTA (Invitrogen), and 100 mM Tris buffer (Avantor). 100 x Trolox solution was made by dissolving 100 mg of Trolox in 0.43 mL methanol (Sigma-Aldrich), 0.345 mL 1 M NaOH, and 3.2 mL of Ultrapure Distilled water.

### Exchange-PAINT Imaging Experiments

Exchange DNA-PAINT imaging was performed on a custom-built total internal reflection fluorescence (TIRF) microscope based on a Nikon Eclipse Ti-2 microscope (Nikon Instruments) equipped with a 100×oil immersion TIRF objective (Apo TIRF, NA 1.49) and a Perfect Focus System. Samples were imaged under flat-top TIRF illumination with a 647 nm laser (Coherent OBIS LX, 120 mW), that was magnified with custom-built telescopes, before passing through a beam shaper device (piShaper 6_6_VIS, AdlOptica) to transform the Gaussian profile of the beam into a collimated flat-top profile. The beam was focused into the back focal plane of the microscope objective using a suitable lens (AC508-300-A-ML, Thorlabs), passed through a clean-up filter (FF01-390/482/563/640-25, Semrock), and coupled into the objective using a beam splitter (Di03-R405/488/561/635-t1-25 × 36, Semrock). Laser polarization was adjusted to circular after the objective. Fluorescence light was spectrally filtered with an emission filter (FF01-446/523/600/677-25, Semrock) and imaged on an sCMOS camera (ORCA-Flash4.0 V3 Digital, Hamamatsu) without further magnification, resulting in a final pixel size of 130 nm in the focal plane, after 2 × 2 binning. For fluid exchange, each individual chamber of the ibidi µ-SlideVI^0.5^ was fitted with elbow Luer connector male adaptors (Ibidi) and 0.5 mm silicon tubing (Ibidi). Each imaging acquisition step was performed by adding the corresponding imager strand buffer solution to the sample. Prior to the imager exchange, the chamber was washed for 10 min with 1 x PBS buffer with 500  mM NaCl. Before the next imager strand buffer solution was added, we monitored with the camera to ensure the complete removal of the first imager strand. Sequential imaging and washing steps were repeated for every cell imaged. For each imaging step, 15,000 frames were acquired with 100ms integration time and a laser power density at the sample of 0.5 kW/cm^2^.

### Super-resolution DNA-PAINT image reconstruction

Both P2Y_2_ and αV Images were processed and reconstructed using the Picasso (Schnitzbauer et al., 2017) software (Version 0.3.3). The Picasso ‘Localize’ module was used to identify and localize the x, y molecular coordinates of single molecule events from the raw fluorescent DNA-PAINT images. Drift correction and multi-color data alignment were performed via the Picasso ‘Render’ module, using a combination of fiducial markers and multiple rounds of image sub-stack cross-correlation analysis. Localizations with uncertainties greater than 13 nm were removed and no merging was performed for molecules re-appearing in subsequent frames. Super-resolution image rendering was performed by plotting each localization as a Gaussian function with a standard deviation equal to its localization precision.

### Protein quantification via qPAINT analysis

To convert the list of *x, y* localizations into a list of *x, y* protein coordinates the data was further processed using a combination of DBSCAN cluster analysis, qPAINT analysis, and *k-*means clustering.

First, 21 randomly selected, non-overlapping, 4 × 4 µm^2^ regions of interest (ROIs) for each type of cell and cell treatment were analyzed with a density-based clustering algorithm, known as DBSCAN. To avoid suboptimal clustering results; ROIs were selected such that they do not intersect with cell boundaries and the regions were the same for P2Y_2_ and αV images. Single-molecule localizations within each ROIs were grouped into clusters using the DBSCAN modality from PALMsiever (Pengo et al., 2015) in MATLAB (Version 2021a)(Pengo et al., 2015). This clustering algorithm determines clusters based on two parameters. The first parameter is the minimum number of points (‘minPts’) within a given circle. For minPts, we chose a parameter in accordance with the binding frequency of the imager strand and acquisition frame number; in our case this was set to 10 localizations for all the experiments. The second parameter is the radius (epsilon or ‘eps’) of the circle of the cluster of single molecule localizations. This is determined by the localization precision of the super-resolved images and, according to the nearest neighbor based analysis was ca. to 10 nm for all the images.

For qPAINT analysis, we used a custom-written MATLAB (Version 2021a) code: https://github.com/Simoncelli-lab/qPAINT_pipeline (Joseph and Simoncelli, 2023). Briefly, localizations corresponding to the same cluster were grouped and their time stamps were used to compile the sequence of dark times per cluster. All the dark times per cluster were pooled and used to obtain a normalized cumulative histogram of the dark times which was then fitted with the exponential function 1 – exp(t/τ_d_) to estimate the mean dark time, τ_d_, per cluster. The qPAINT index (*q*_i_) of each cluster was then calculated as the inverse of the mean dark time, 1/τ_d_.

Calibration was then performed via a compilation of all qPAINT indexes obtained from the DNA-PAINT data acquired for each protein type into a single histogram. Only qPAINT indices corresponding to small clusters (i.e. clusters with a maximum point distance of 150 nm) were considered. This histogram was fitted with a multi-peak Gaussian function to determine the qPAINT index for a cluster of single molecule localizations corresponding to one protein (*q*_i1_).

The calibration value obtained with this method was used to estimate the number of P2Y_2_ and αV proteins in all the single-molecule localizations clusters identified by DBSCAN, as this corresponds to the ratio between *q*_i1_ and the qPAINT index of each cluster. Finally, *k*-means clustering was used to recover a likely distribution of the proteins’ positions in each cluster of single molecule localizations, where *k* is equal to the number of proteins in that cluster. This information allowed us to quantify the protein density and level of protein clustering.

### Nearest neighbor analysis

Nearest neighbor distances (NND) for P2Y_2_ – P2Y_2_ and αV-αV were calculated using the recovered P2Y_2_ and αV-protein maps as described above via a custom-written MATLAB (Version 2021a) script: https://github.com/Simoncelli-lab/qPAINT_pipeline (Joseph and Simoncelli, 2023). For colocalization analysis, the NND for each protein of one dataset with respect to the reference dataset was calculated (i.e. P2Y_2_ - αV) using a similar MATLAB script. To evaluate the significance of the NND distributions, we randomized the positions of P2Y_2_ and αV for the comparison of P2Y_2_ – P2Y_2_ and αV-αV NND distributions, respectively, and the positions of one of the two proteins for the comparison of the NND between P2Y_2_ - αV protein distributions. The resulting histogram of the nearest neighbor distances for both the experimental data sets and the randomly distributed data was normalized using the total number of NND calculated per ROI to calculate the percentage of the population with distances smaller than a set threshold value.

### Western blotting

Cell lysates were extracted using RIPA buffer and 20 µg denatured protein per sample was loaded and separated using an 8% SDS-PAGE gel. Gels were run at 150 V for 2 hr and transferred into a nitrocellulose membrane (GE Healthcare) at 100 V for 1 hr. Following blocking with 5% milk (Sigma) in 0.1% TBS-T for 1 hr, membranes were incubated with 1:1000 dilution of antibodies against phosphorylated FAK (Tyr397, 3283, Cell Signaling, RRID:AB_2173659), phosphorylated ERK 1/2 (S217/221, 9154, Cell Signaling, RRID:AB_2138017), P2Y_2_ (APR-010, Alomone Labs, RRID:AB_2040078), HSC 70 (SC7298, Santa Cruz, RRID:AB_627761), or α-tubulin (T5168, Sigma-Aldrich, RRID:AB_477579) with 5% BSA in 0.1% TBS-T overnight at 4 °C. Membranes were probed with anti-Mouse-HRP (P0447, DAKO, RRID:AB_2617137), or Anti-Rabbit-HRP (P0448, DAKO, RRID:AB_2617138) at 1:5000 in 5% milk in TBS-T for 1 hr at room temperature. Images were captured by using Luminata Forte Western HRP substrate (Millipore) and imaged with an Amersham Imager 600 (GE Healthcare).

### Statistical analysis

For the statistical analysis of the number and colocalization of DNA-PAINT images, a minimum of five 4 × 4 µm^2^ regions obtained from AsPC-1 cells were analyzed per condition. For all experiments, normality tests were performed and the non-parametric Kruskal-Wallis test for significance was calculated. All graphs and statistical calculations of experimental data were made using Prism 9.4.1 (GraphPad).

## Data Availability

All data generated or analysed during this study are included in the manuscript and supporting file, or online resources are fully referenced. Human PDAC tumour data were generated by TCGA Research Network (https://www.cancer.gov/tcga) and by the Clinical Proteomic Tumour Analysis Consortium (https://www.proteomics.cancer.gov). The Genotype-Tissue Expression (GTEx) Project was used for the analysis of normal pancreatic tissue samples (https://gtexportal.org).

## Additional files

Supplementary file 1. Pancreatic cancer molecular subtypes associated with purinergic gene expressions. Purinergic genes with significantly higher expression in a specific molecular subtype have been listed below. If no significant higher expression was observed not applicable (N/A) is shown.

MDAR checklist
