# Supplementary figures and images for: Purinergic GPCR-integrin interactions drive pancreatic cancer cell invasion
Source: eLife. 2023 Mar 21;12:e86971. doi: 10.7554/eLife.86971 (PMC10069867; doi:10.7554/eLife.86971)

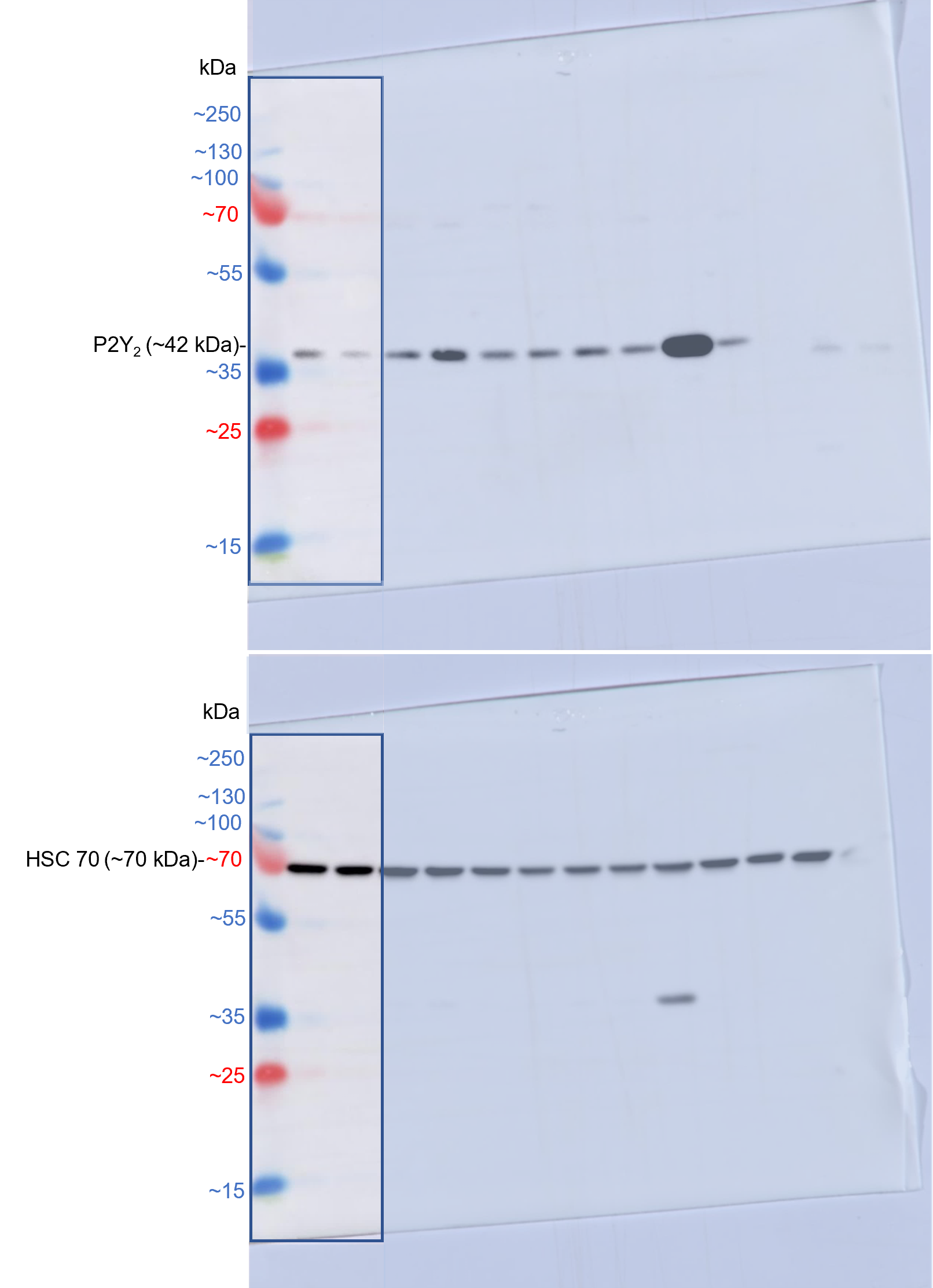

Supplement: Figure 3—figure supplement 1—source data 1. [file elife-86971-fig3-figsupp1-data1.zip › Figure 3- figure supplement 1I-labelled uncropped blot.png]

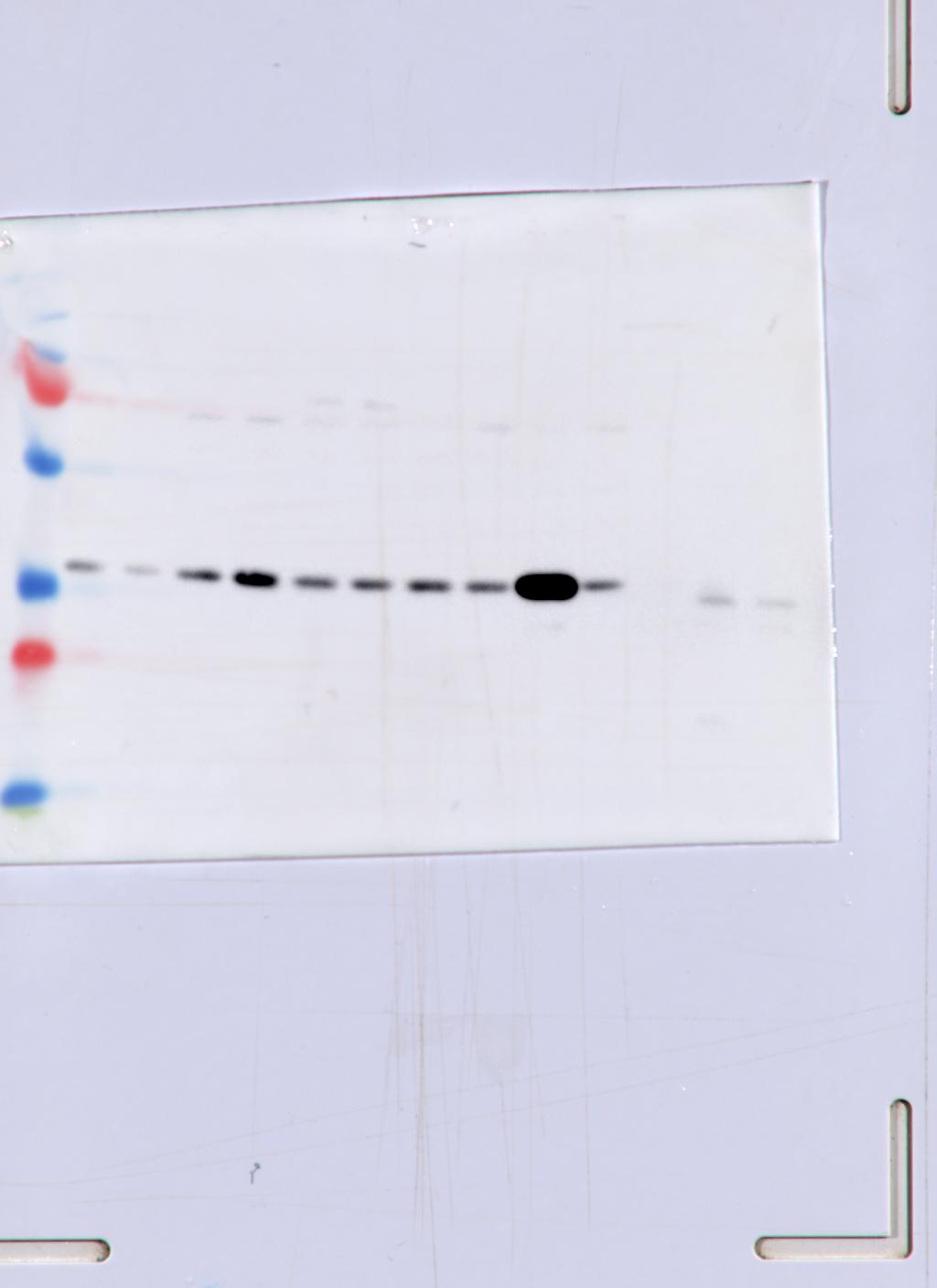

Supplement: Figure 3—figure supplement 1—source data 2. [file elife-86971-fig3-figsupp1-data2.zip › Figure 3-figure supplement 1I-raw P2Y2.jpg]

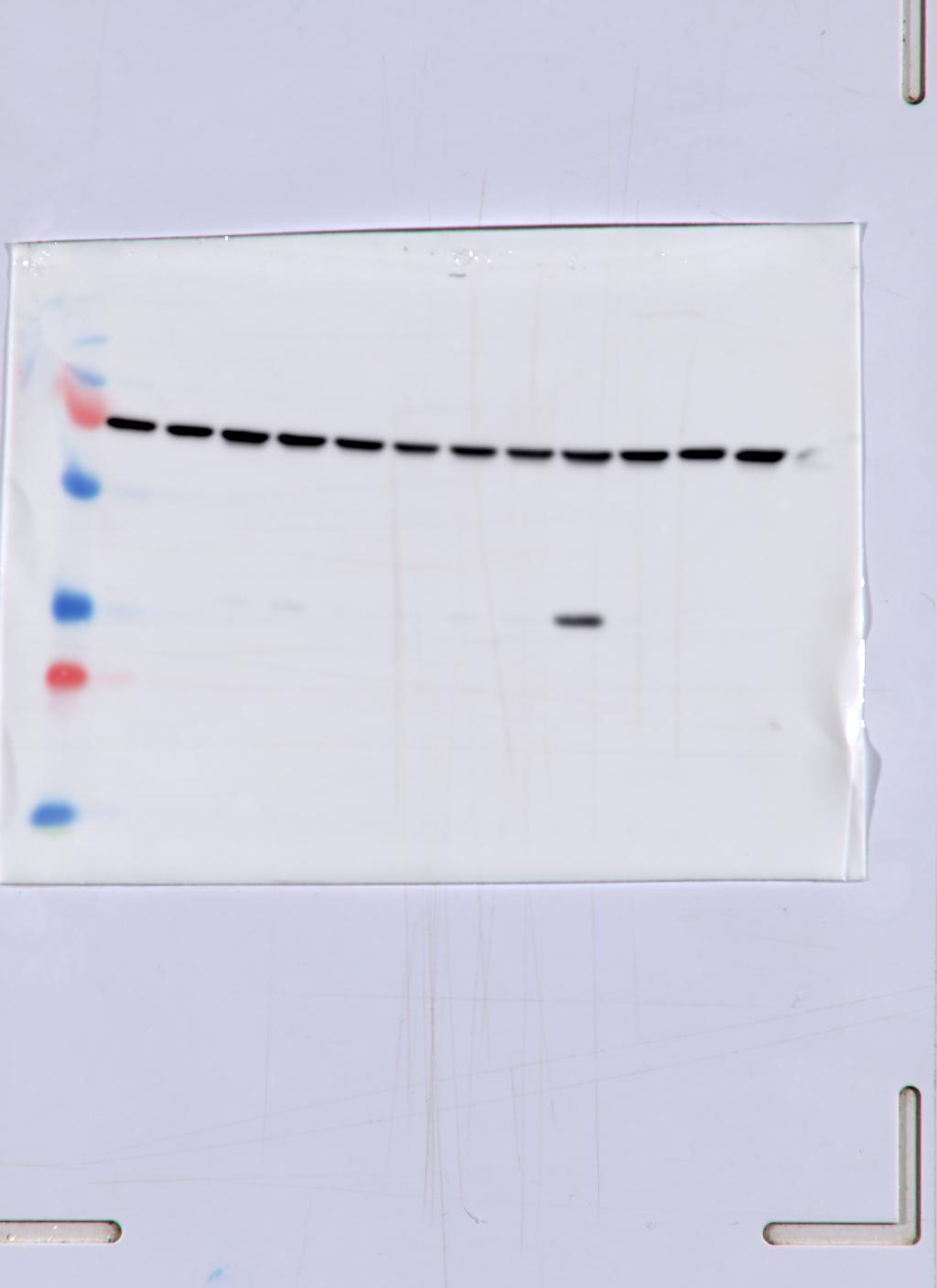

Supplement: Figure 3—figure supplement 1—source data 2. [file elife-86971-fig3-figsupp1-data2.zip › Figure 3-figure supplement 1I-raw HSC 70.jpg]

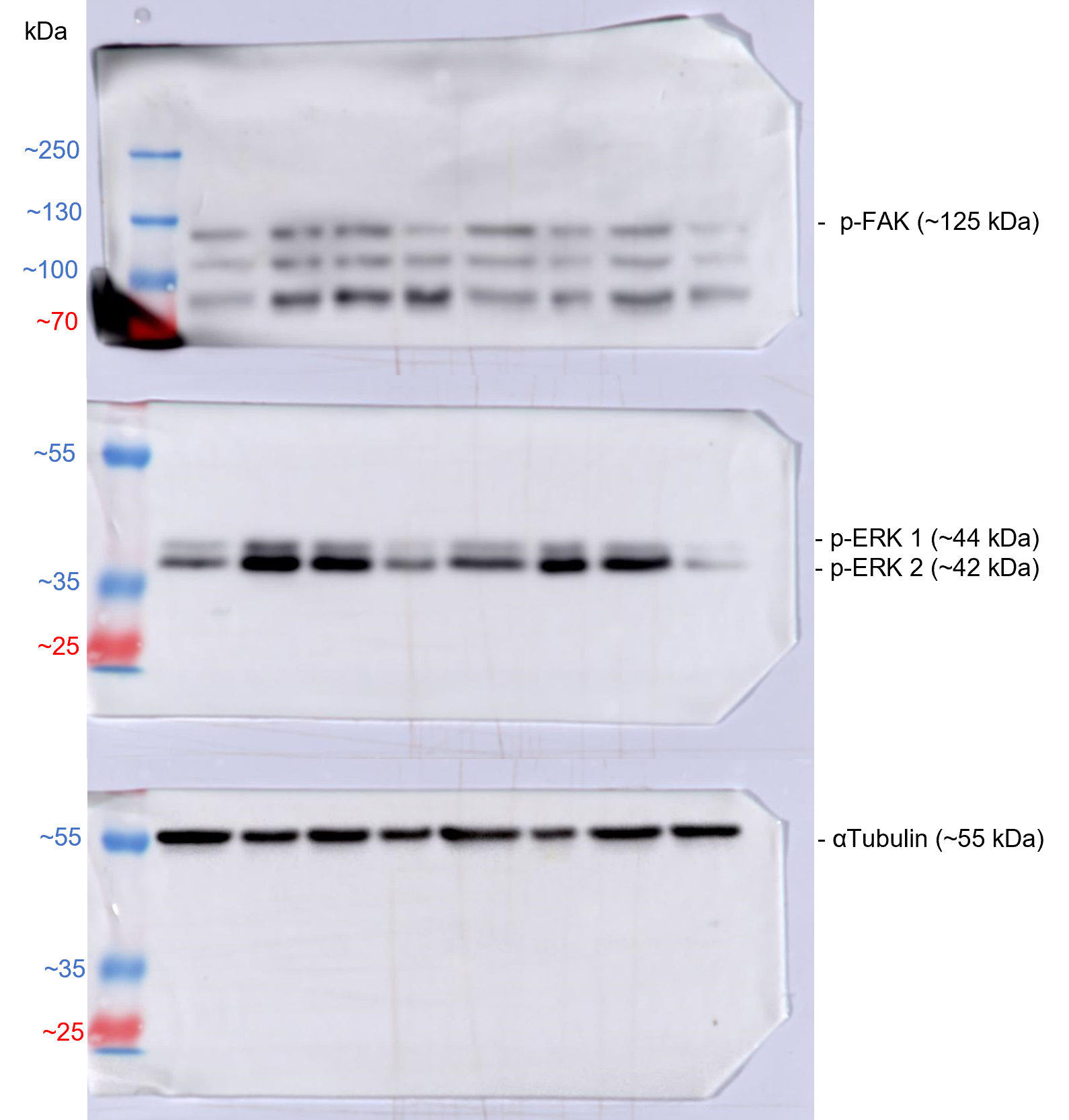

Supplement: Figure 5—source data 1. [file elife-86971-fig5-data1.zip › Figure 5 C-labelled uncropped blot.png]

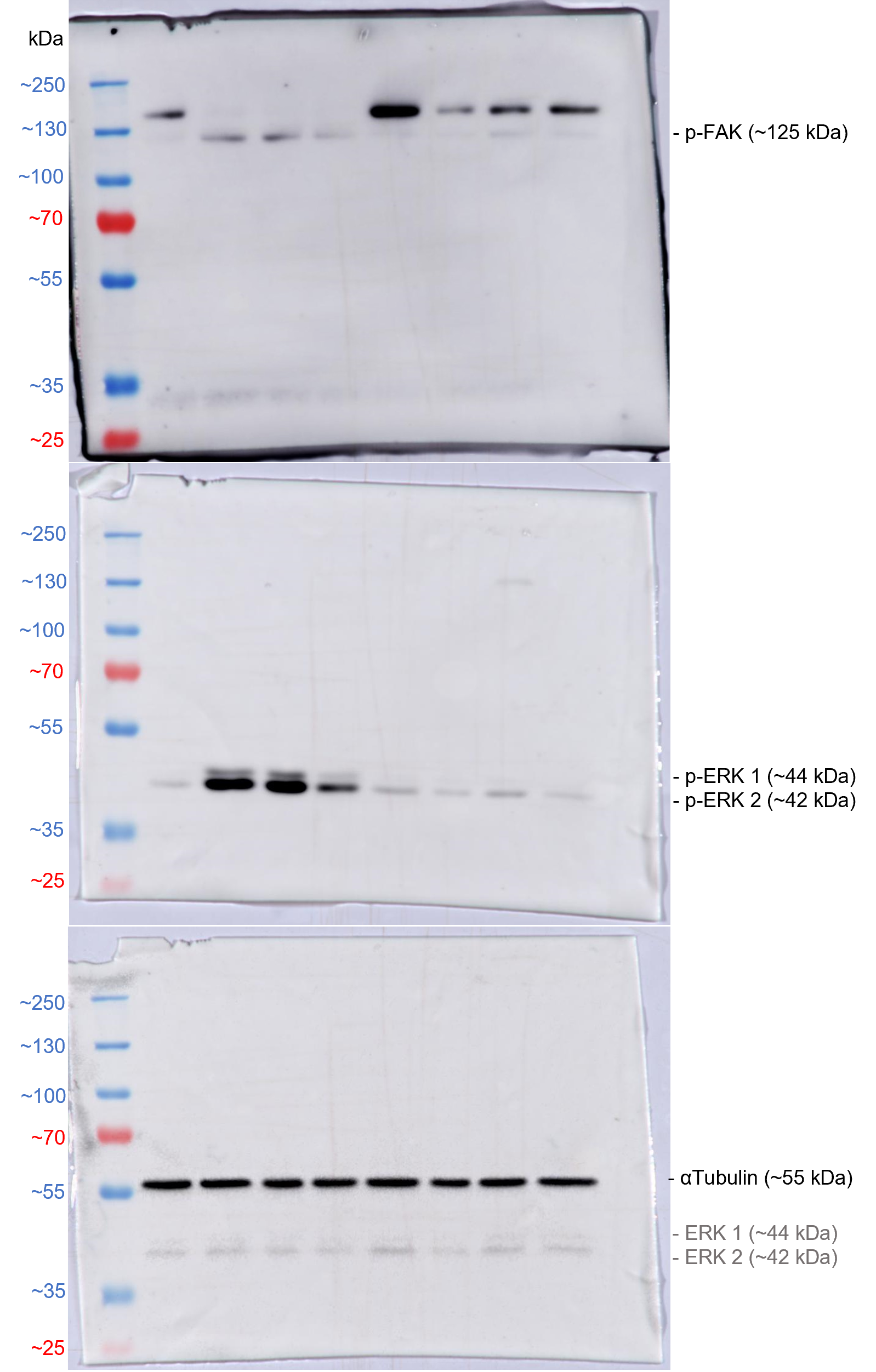

Supplement: Figure 5—source data 1. [file elife-86971-fig5-data1.zip › Figure 5 A-labelled uncropped blot.png]

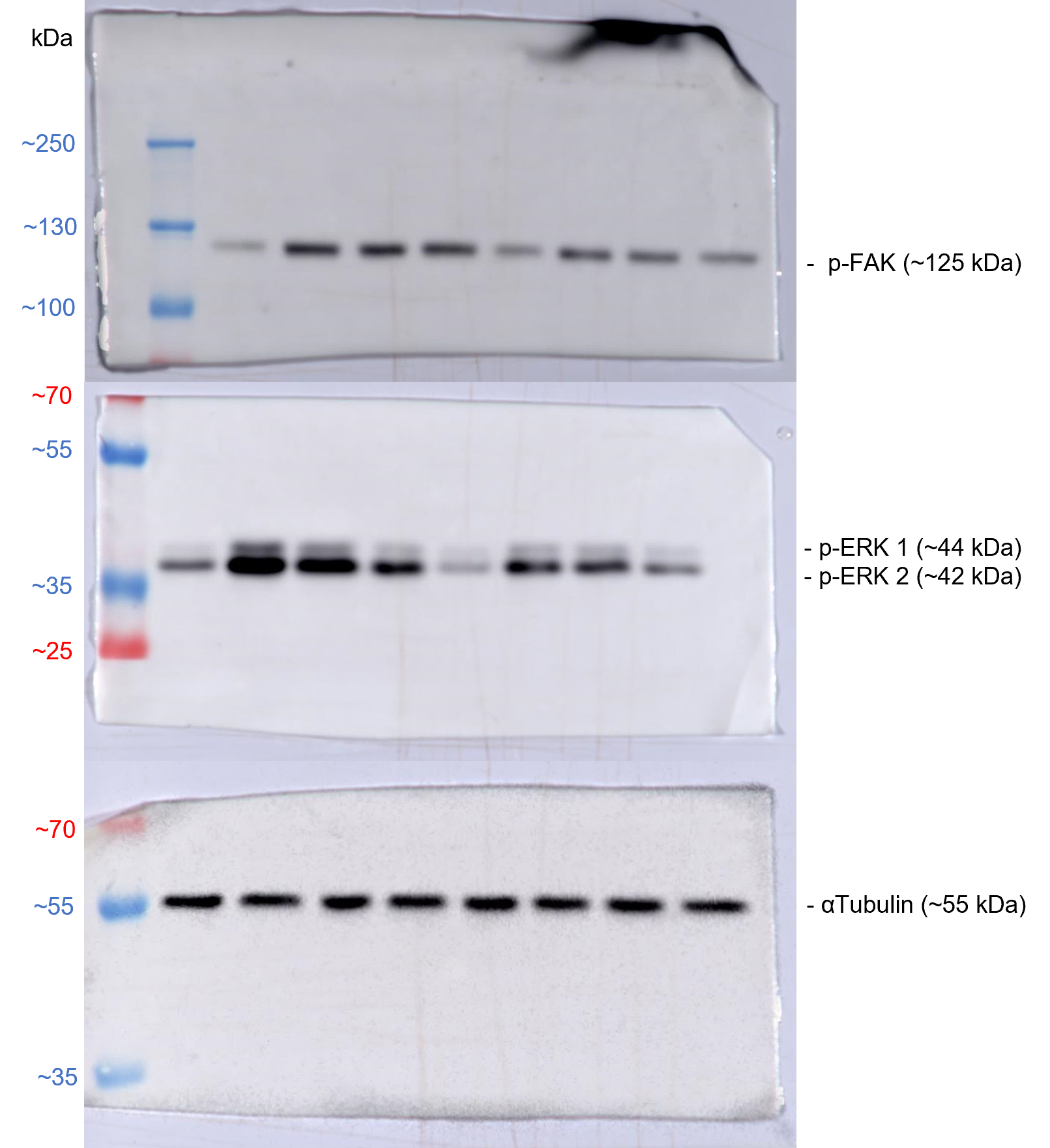

Supplement: Figure 5—source data 1. [file elife-86971-fig5-data1.zip › Figure 5 B-labelled uncropped blot.png]

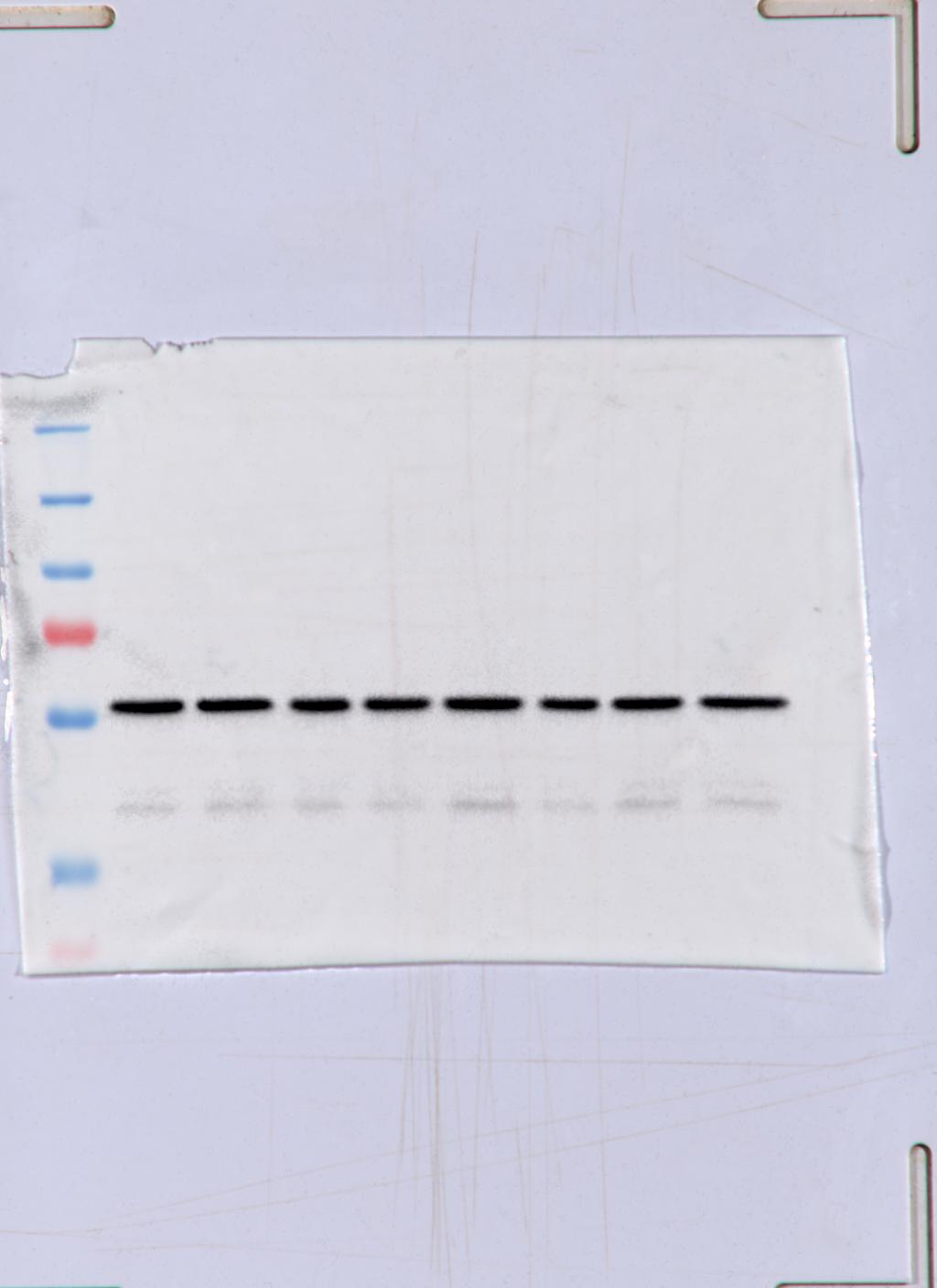

Supplement: Figure 5—source data 2. [file elife-86971-fig5-data2.zip › Figure 5A raw alphaTubulin.jpg]

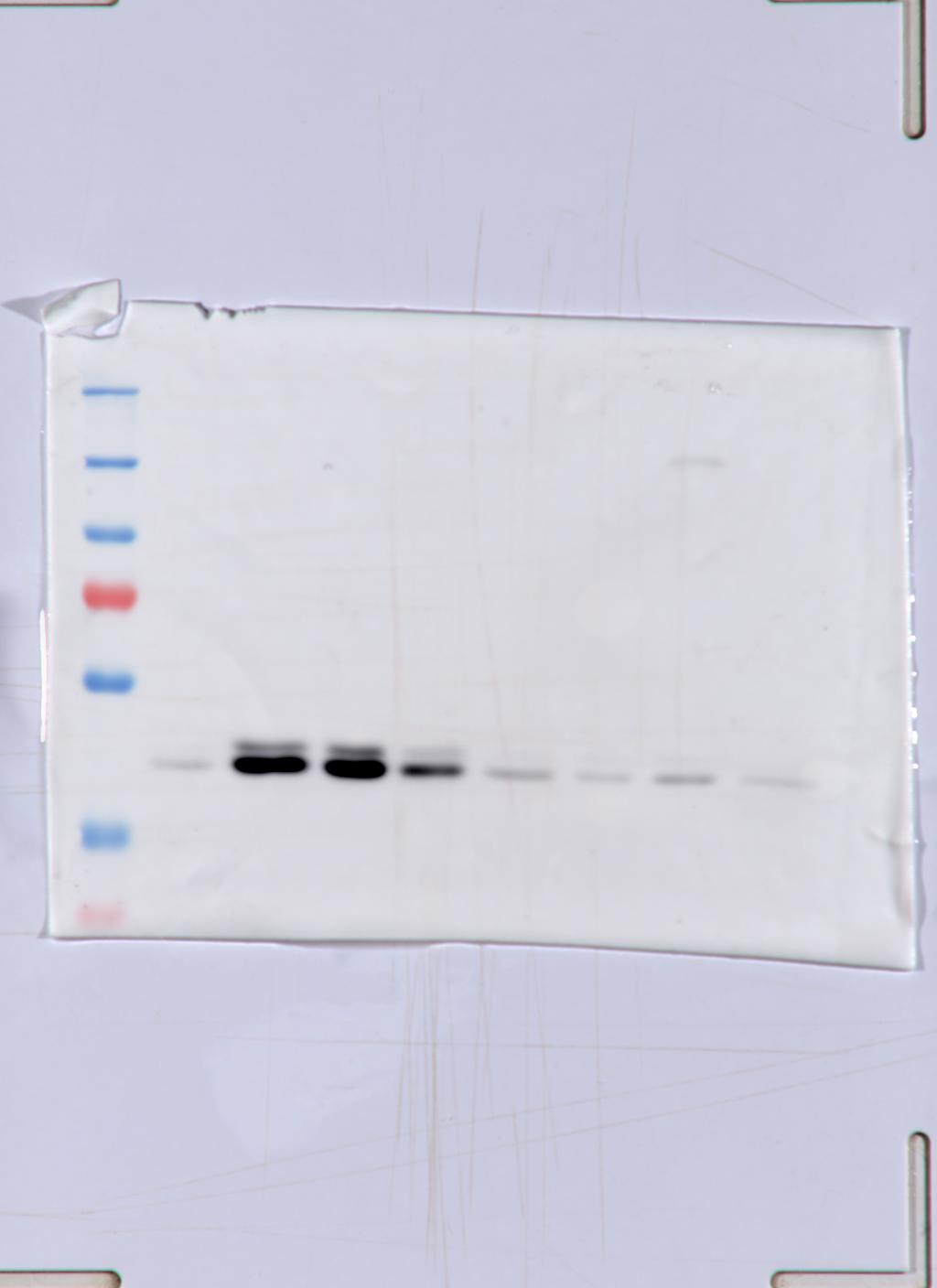

Supplement: Figure 5—source data 2. [file elife-86971-fig5-data2.zip › Figure 5A raw p-ERK.jpg]

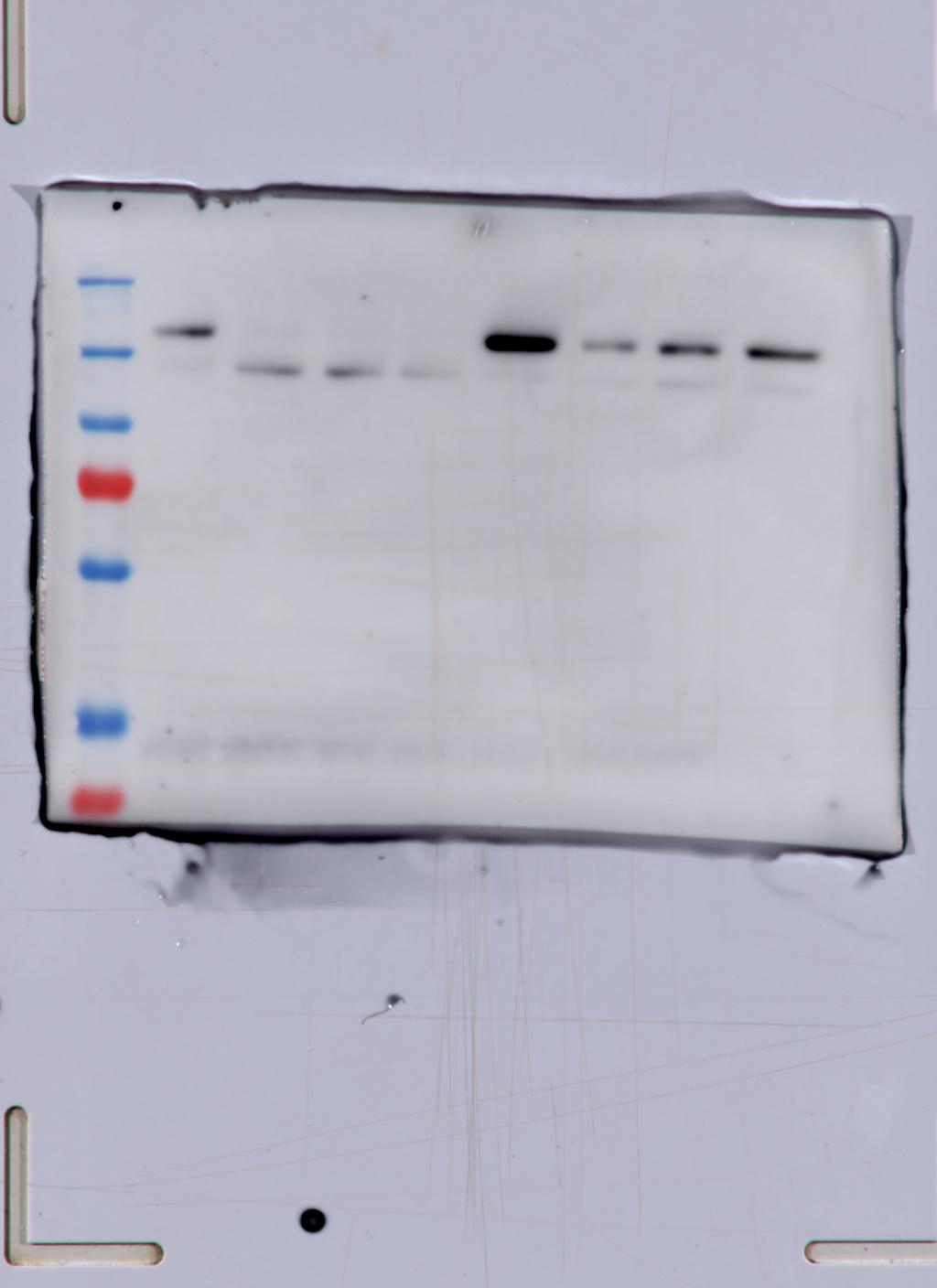

Supplement: Figure 5—source data 2. [file elife-86971-fig5-data2.zip › Figure 5A raw p-FAK.jpg]

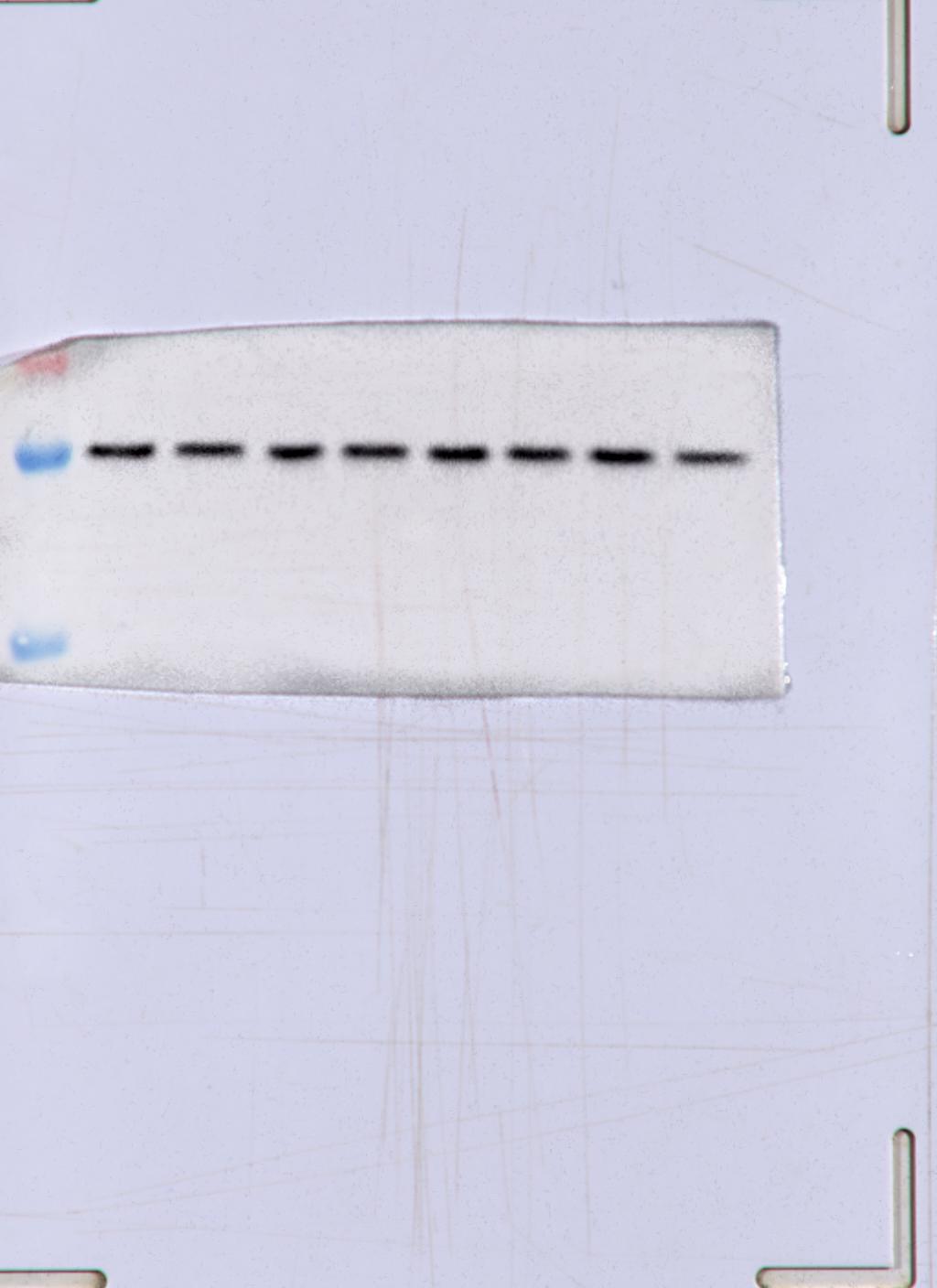

Supplement: Figure 5—source data 2. [file elife-86971-fig5-data2.zip › Figure 5B raw alphaTubulin.jpg]

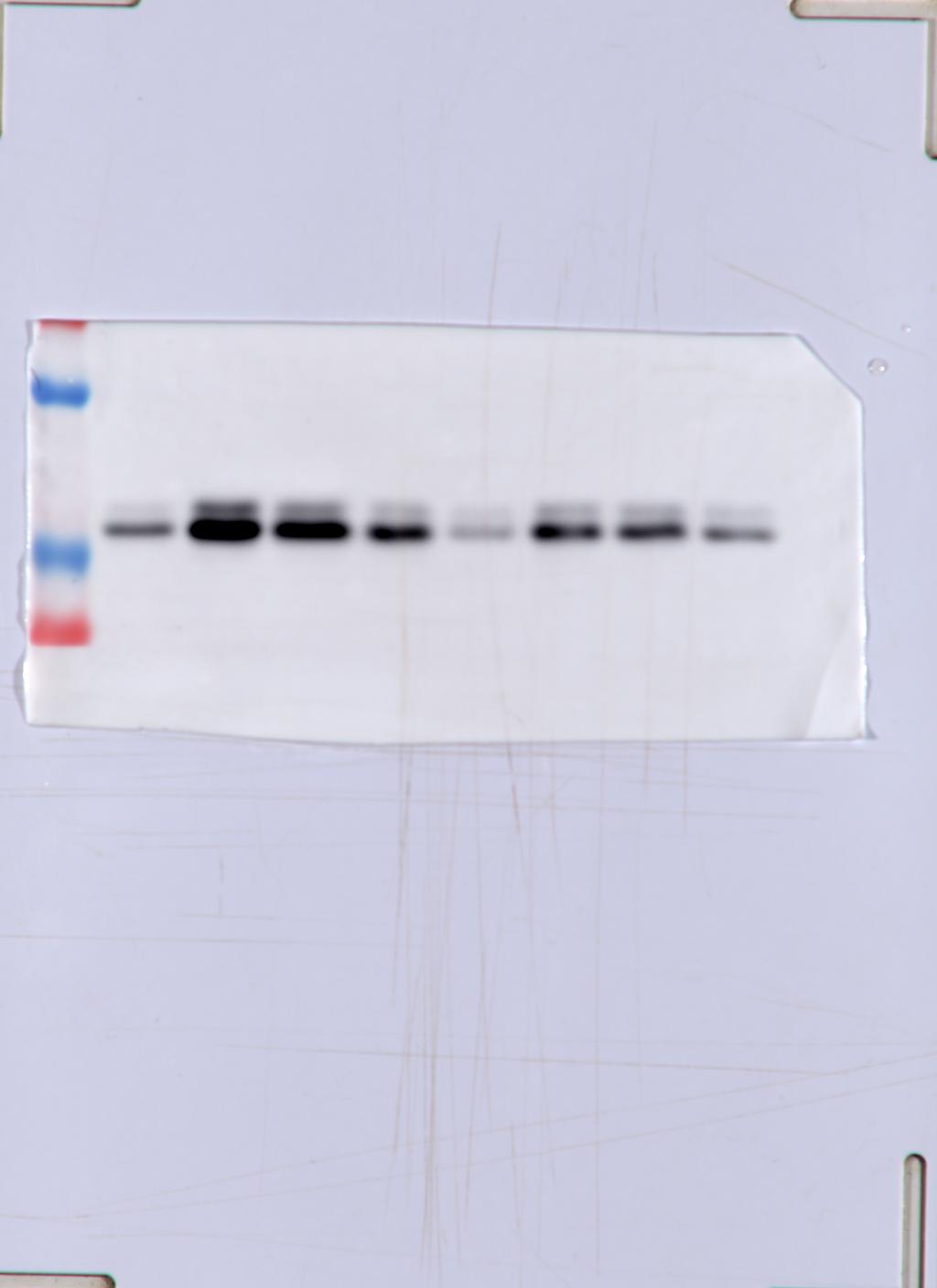

Supplement: Figure 5—source data 2. [file elife-86971-fig5-data2.zip › Figure 5B raw p-ERK.jpg]

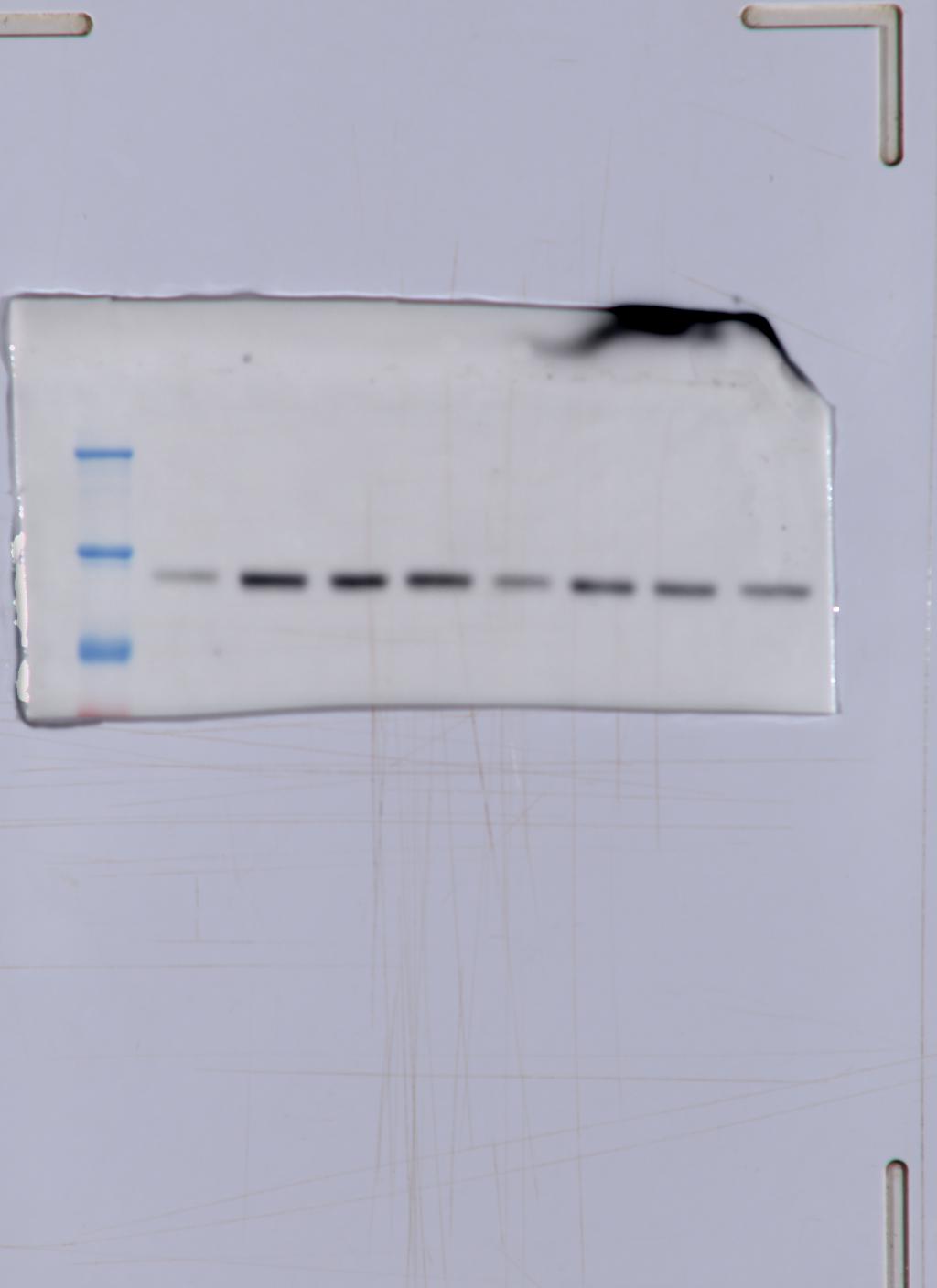

Supplement: Figure 5—source data 2. [file elife-86971-fig5-data2.zip › Figure 5B raw p-FAK.jpg]

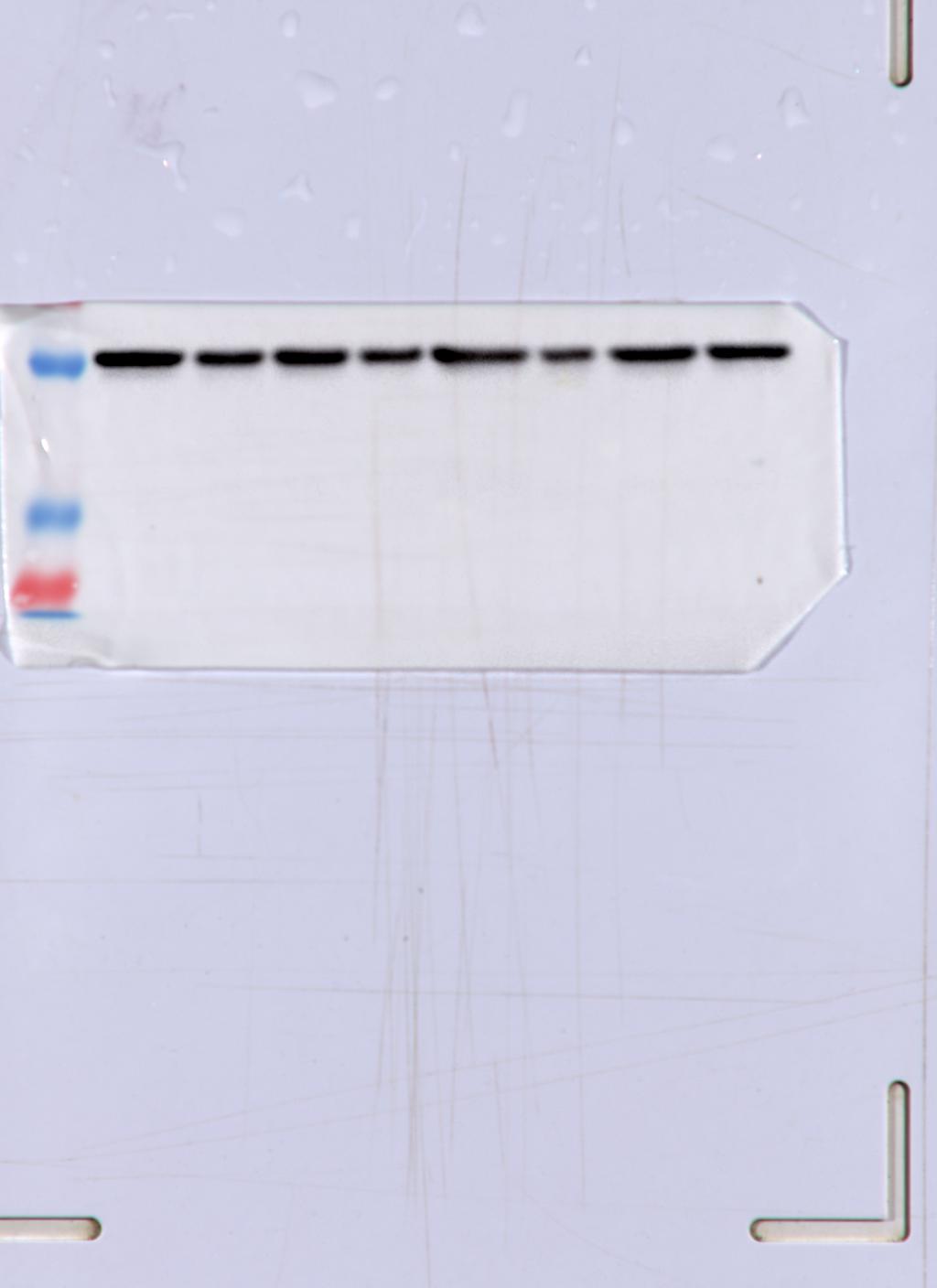

Supplement: Figure 5—source data 2. [file elife-86971-fig5-data2.zip › Figure 5C raw alphaTubulin.jpg]

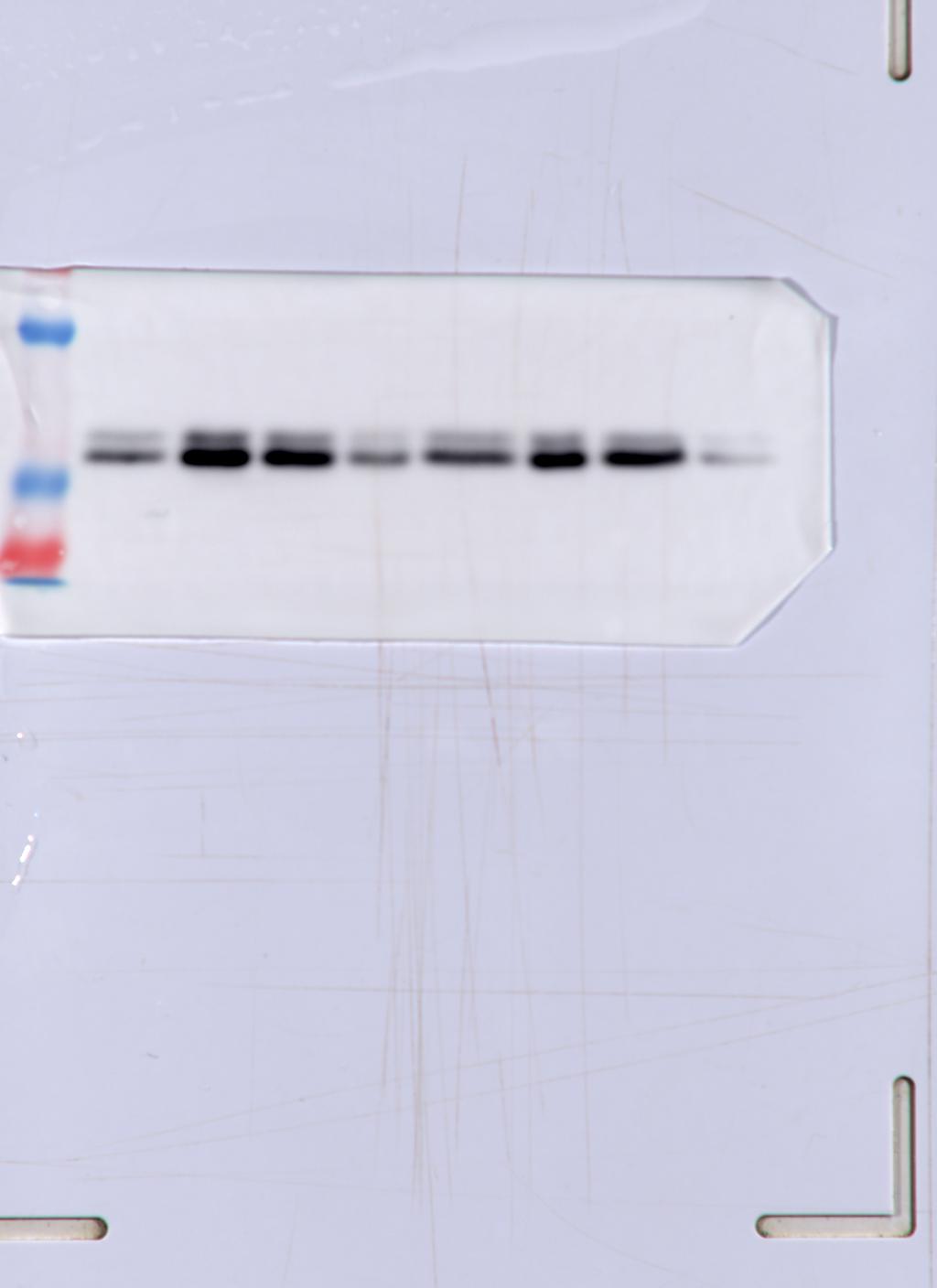

Supplement: Figure 5—source data 2. [file elife-86971-fig5-data2.zip › Figure 5C raw p-ERK.jpg]

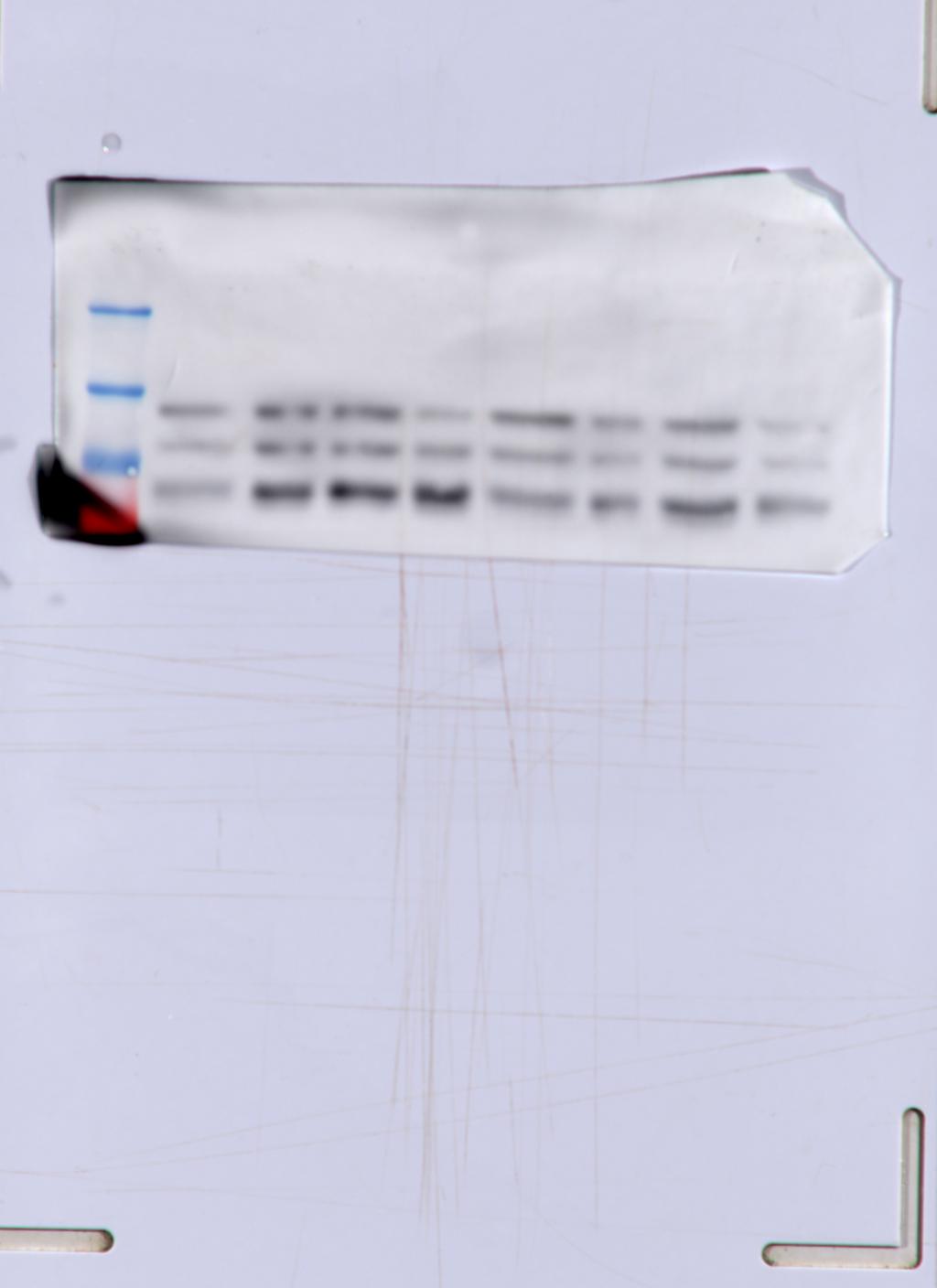

Supplement: Figure 5—source data 2. [file elife-86971-fig5-data2.zip › Figure 5C raw p-FAK.jpg]
