# Supplementary material for: Purinergic GPCR-integrin interactions drive pancreatic cancer cell invasion
Source: eLife. 2023 Mar 21;12:e86971. doi: 10.7554/eLife.86971 (PMC10069867; doi:10.7554/eLife.86971)
Supplement: Supplementary file 1. — Purinergic genes with significantly higher expression in a specific molecular subtype have been listed below. If no significant higher expression was observed not applicable (N/A) is shown. [file elife-86971-supp1.docx]

| **Genes** | **Bailey** | **Collisson** | **Moffitt** |
| --- | --- | --- | --- |
| ***ADA*** | Immunogenic | Quasimesenchymal | Basal |
| ***NT5E*** | Squamous | Quasimesenchymal | Basal |
| ***ENTPD1*** | Immunogenic | Quasimesenchymal | N/A |
| ***ADORA3*** | Immunogenic | Exocrine | N/A |
| ***ADORA2B*** | Squamous | Classical | N/A |
| ***ADORA2A*** | Immunogenic | Quasimesenchymal | N/A |
| ***ADORA1*** | ADEX | Exocrine | N/A |
| ***P2RX7*** | Immunogenic | Exocrine | N/A |
| ***P2RX6*** | Immunogenic | N/A | N/A |
| ***P2RX5*** | Immunogenic | Quasimesenchymal | N/A |
| ***P2RX4*** | ADEX | Exocrine | N/A |
| ***P2RX3*** | N/A | N/A | N/A |
| ***P2RX2*** | Immunogenic | N/A | N/A |
| ***P2RX1*** | ADEX | Exocrine | N/A |
| ***P2RY14*** | Immunogenic | Quasimesenchymal | N/A |
| ***P2RY13*** | Immunogenic | Exocrine | N/A |
| ***P2RY12*** | Immunogenic | Exocrine | N/A |
| ***P2RY11*** | ADEX | N/A | N/A |
| ***P2RY10*** | Immunogenic | Quasimesenchymal | N/A |
| ***P2RY8*** | Immunogenic | Quasimesenchymal | N/A |
| ***P2RY6*** | Immunogenic | Quasimesenchymal | Basal |
| ***P2RY4*** | N/A | N/A | N/A |
| ***P2RY2*** | Squamous | N/A | Basal |
| ***P2RY1*** | ADEX | Exocrine | N/A |
| ***PANX1*** | Squamous | Quasimesenchymal | Basal |

**Supplementary file 1. Pancreatic cancer molecular subtypes associated with purinergic gene expressions.** Purinergic genes with significantly higher expression in a specific molecular subtype have been listed below. If no significant higher expression was observed not applicable (N/A) is shown.
